# A BMP-FGF Morphogen Toggle Switch Drives the Ultrasensitive Expression of Multiple Genes in the Developing Forebrain

**DOI:** 10.1371/journal.pcbi.1003463

**Published:** 2014-02-13

**Authors:** Shyam Srinivasan, Jia Sheng Hu, D. Spencer Currle, Ernest S. Fung, Wayne B. Hayes, Arthur D. Lander, Edwin S. Monuki

**Affiliations:** 1 Department of Developmental and Cell Biology, University of California, Irvine, California, United States of America; 2 Center for Complex Biological Systems, University of California, Irvine, California, United States of America; 3 Department of Computer Science, University of California, Irvine, California, United States of America; 4 Department of Developmental Neurobiology, St Jude Children's Research Hospital, Memphis, Tennessee, United States of America; 5 Department of Pathology and Laboratory Medicine, University of California, Irvine, California, United States of America; Princeton University, United States of America

## Abstract

Borders are important as they demarcate developing tissue into distinct functional units. A key challenge is the discovery of mechanisms that can convert morphogen gradients into tissue borders. While mechanisms that produce ultrasensitive cellular responses provide a solution, how extracellular morphogens drive such mechanisms remains poorly understood. Here, we show how Bone Morphogenetic Protein (BMP) and Fibroblast Growth Factor (FGF) pathways interact to generate ultrasensitivity and borders in the dorsal telencephalon. BMP and FGF signaling manipulations in explants produced border defects suggestive of cross inhibition within single cells, which was confirmed in dissociated cultures. Using mathematical modeling, we designed experiments that ruled out alternative cross inhibition mechanisms and identified a cross-inhibitory positive feedback (CIPF) mechanism, or “toggle switch”, which acts upstream of transcriptional targets in dorsal telencephalic cells. CIPF explained several cellular phenomena important for border formation such as threshold tuning, ultrasensitivity, and hysteresis. CIPF explicitly links graded morphogen signaling in the telencephalon to switch-like cellular responses and has the ability to form multiple borders and scale pattern to size. These benefits may apply to other developmental systems.

## Author Summary

During development, morphogen gradients play a crucial role in transforming a uniform field of cells into regions with distinct cell identities (marked by the expression of specific genes). Finding mechanisms that convert morphogen gradients into sharp borders of gene expression, however, remains a challenge. Cellular ultrasensitivity mechanisms that convert a linear stimulus into an on-off target response offer a good solution for making such borders. In this paper, we show how a cross-inhibitory positive feedback or toggle switch mechanism driven by two extracellular morphogens – BMP and FGF - produces ultrasensitivity in forebrain cells. Experiments with cells and explanted brain tissue reveal that BMPs and FGFs cross inhibit each other's signaling pathway. Such cross inhibition could occur through four possible mechanisms. By an iterative combination of modeling and experiment, we show the toggle switch to be the mechanism underlying cross inhibition, the ultrasensitive expression of multiple genes, and hysteresis in forebrain cells. As the toggle switch explicitly links extracellular morphogens to cellular ultrasensitivity, it provides a mechanism for making multiple sharp borders that can also scale with tissue size – an important issue in pattern formation. This might explain the abundance of BMP-FGF cross inhibition during development.

## Introduction

The formation of borders between compartments and body parts is crucial for embryonic development [1], [2], [3], [4], [5]. A challenge in understanding border formation is the elucidation of mechanisms that convert shallow morphogen gradients into sharp expression domains [3],[6]. Such mechanisms fall into two categories: those that involve cell-cell cooperation, such as cell sorting [2], [5], and those that do not and are therefore cell-intrinsic. Cell-intrinsic border-forming mechanisms amplify small fold-changes in extracellular morphogen concentration into large fold-changes in target gene expression [7]. Such ‘switch-like’ behavior, also known as ultrasensitivity, enables cells embedded in a morphogen gradient to convert slight differences in morphogen concentration into sharp gene expression domains.

Extensive studies in many systems [6], including the mammalian spinal cord [8] and syncytial fly blastoderm [6], [9], show that ultrasensitivity and border formation can result from complex interactions between a morphogen and its downstream transcription factor network, or within a transcriptional network alone. While such morphogen-transcription networks have been explored, the interactions between extracellular morphogens as a basis for ultrasensitivity has not been described, even though such interactions are common in development [10].

One system patterned by interacting morphogens is the dorsal telencephalon [11], in which cell-intrinsic ultrasensitivity was proposed to mediate border formation between the telencephalic dorsal midline (DM) and cerebral cortex [12]. The DM - located between the cerebral cortices – develops from the roof plate and adjacent tissues to form the choroid plaque, choroid plexus epithelium (CPE), and cortical hem [13] along the mediolateral axis. These tissues produce BMPs - including BMP4 - at high levels [14] to form an activity gradient of BMP signaling [12], [15], with BMP-dependent genes *Msx1* and *Ttr* being expressed in the CPE [15], where BMP activity is highest. *Msx1* is a high-threshold BMP target gene in many patterning systems [16], [17], including the dorsal telencephalon [12], [13], [14], while *Ttr* is induced specifically in the CPE at the onset of its definitive differentiation (∼embryonic day 11, or E11, in mice) and is stably expressed thereafter [18].

Although *Msx1* is restricted to the midline, BMP4 can induce *Msx1* expression in dissociated cortical precursor cells (CPCs) in an ultrasensitive fashion [12]. Both *in vivo* and *in vitro*, *Msx1* ultrasensitivity contrasts with graded changes in nuclear phospho-Smad1, 5, or 8 (pSmad) levels (a direct readout of BMP signaling intensity), within the same cells. This implies that *Msx1* ultrasensitivity occurs downstream of pSmad activation. The mechanism underlying this ultrasensitivity, however, remains unknown.

Dorsal telencephalic cells responsive to BMP also respond to other morphogens, such as FGFs (most notably FGF8) produced in the adjacent rostral midline (RM) and cortex [11], [19], [20], [21], [22], [23]. FGF8 in the RM functions as a graded morphogen [24], and in the chick dorsal forebrain negatively regulates BMP target genes by inhibiting dorsal BMP4 expression [25], [26]. In other systems, FGFs inhibit BMP signaling through MAPK-mediated phosphorylation of Smads [27], [28].

We investigated the influence of FGFs on DM BMP target genes, and found that ultrasensitivity requires cell-intrinsic interactions between the BMP and FGF pathways. Using explants and dissociated cell cultures, we showed that the BMP and FGF pathways mutually inhibit at the single cell level; Epidermal Growth Factor (EGF) acts similarly to FGF. Next, we used modeling to identify experiments that distinguish among different models of cross-inhibition. These experiments identified a cross-inhibitory positive feedback (CIPF) mechanism, or “toggle-switch”, between the BMP and FGF signaling pathways as the basis for ultrasensitivity. We further show how this mechanism is capable of generating multiple sharp borders simultaneously, among other potential advantages.

## Results

### Exogenous BMP4 upregulates *Ttr* and *Msx1* in midline and cortical cells, respectively

**Figure 1:**
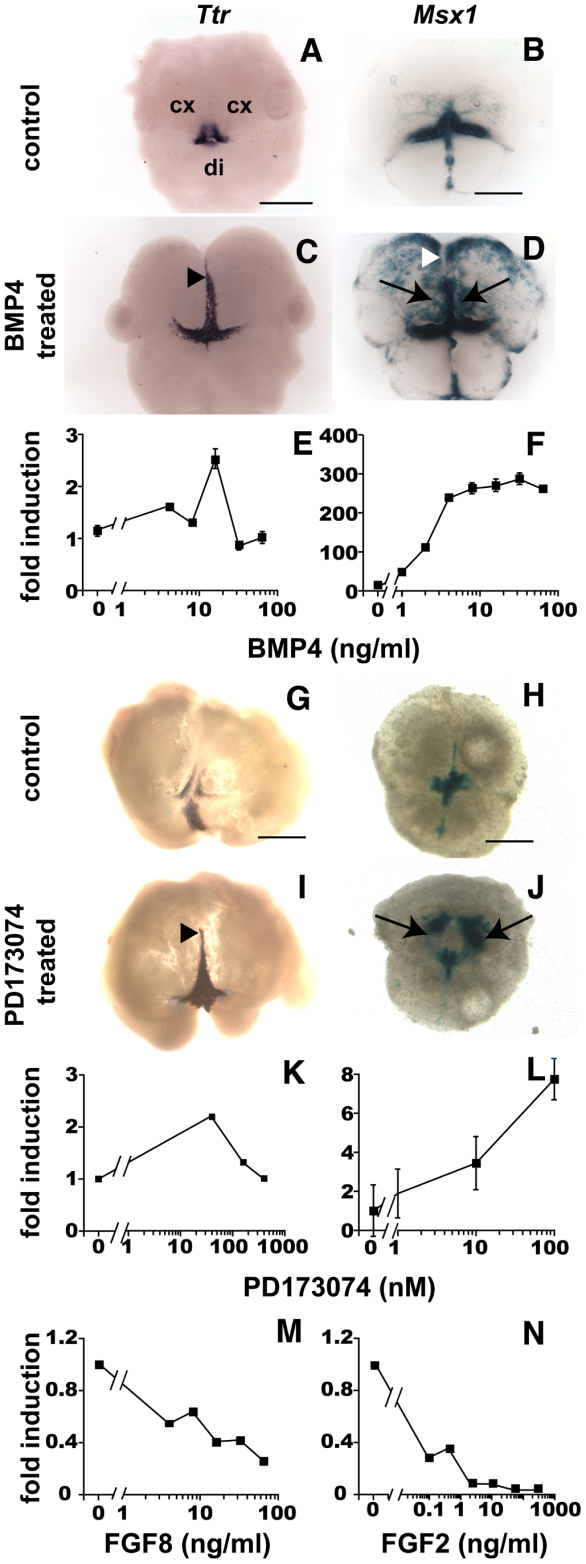
BMP4 upregulates DM genes, while FGFs downregulate them. (**A,C**) *Ttr in situ* hybridization of E9.5 explants treated with or without BMP4 for 72 hrs. Rostral *Ttr* extension (arrowhead in C) was observed in BMP4-treated explants (n = 19/23), but not in BSA-treated controls (n = 0/7). (**B,D**) X-gal stains of E10.5 *Msx1-nlacZ* explants treated with or without BMP4. Ectopic *Msx1-nlacZ* induction occurred in cortex (arrows), but not along the midline (arrowhead in D), in BMP4-treated explants (n = 5/5). Ectopic expression was not detected in BSA-treated controls (n = 0/4). (**E,F**) Dissociated E12.5 midline cells (E) or CPCs (F) assayed for *Ttr* or *Msx1* mRNA levels using RT-qPCR. BMP4 upregulates *Ttr* in midline cells (peak at 16 ng/ml), and monotonically increases *Msx1* in CPCs. (**G–J**) *Ttr* mRNA (G,I) and *Msx1-nlacZ* expression (H,J) in E10.5 explants treated with or without the FGFR inhibitor, PD173704. Like BMP4, PD173704 leads to rostral *Ttr* expression along the midline (arrowhead in I; n = 4/6) compared to controls (DMSO-treated; n = 0/4), as well as *Msx1-nlacZ* induction in cortex (arrows in J), but not the rostral midline (n = 7/8). (**K,L**) Dissociated E12.5 midline cells (K) or CPCs (L) assayed for *Ttr* or *Msx1* mRNA levels by RT-qPCR. PD173074 upregulates *Ttr* levels in midline cells (peak at 64 nM) and monotonically increases *Msx1* levels in CPCs. (**M,N**) Dissociated E12.5 midline cells (M) or CPCs (N) treated with FGF8 or FGF2. FGF8 and FGF2 downregulate *Ttr* and *Msx1* mRNA levels, respectively, in a dose-dependent manner. Abbreviations: di, diencephalon; cx, cortex. Scale bars: 0.5 mm. Error bars represent s.e.m. See also Figure S1.

To experimentally study *Ttr* and *Msx1* regulation in the dorsal telencephalon, we used two previously-characterized *in vitro* systems: a dorsal forebrain explant system and dissociated cultures [12], [15], [29]. First, we treated dorsal forebrain explants with BMP4. E9.5 explants cultured with BMP4 exhibited marked expansion of *Ttr* expression towards the RM (n = 19/23 compared to 0/12 BSA-treated controls; Figure 1A,C). The *Ttr* induction was restricted to the midline, with no expression seen laterally in the cortex. Sections revealed *Ttr* induction in cells lining the ventricular surface (1–2 cell diameters deep), with the *Ttr*-expressing cells often bending inward towards the ventricle (Figure S1C); these features are characteristic of endogenous CPE. Correspondingly, RT-qPCR analysis revealed that CPE and DM marker gene *Lmx1a* was also upregulated rostrally in BMP4-treated explants (Figure S1B). Similar findings were obtained from E10.5 explants (n = 14/18 BMP4-treated, n = 0/6 BSA-treated), although midline *Ttr* induction was more patchy (data not shown).

Interestingly, in response to exogenous BMP4, *Msx1* was ectopically induced in the cortex (n = 5/5 compared to 0/4 BSA-treated explants; Figure 1B,D), but not rostrally in the RM. Thus *Msx1'*s ectopic induction to exogenous BMP4 differs from *Ttr'*s response, which ectopically expands rostrally towards the RM, but not laterally into the cortex.

To determine whether these BMP4-mediated responses are cell-intrinsic, we applied BMP4 to dissociated midline cells and CPCs. Midline cultures included cells from dorsal and rostral regions, as both regions were clearly competent for *Ttr* induction (Figure 1A,C). Both *Ttr* in midline cells and *Msx1* in CPCs were positively regulated by exogenous BMP4 in a concentration-dependent fashion. In midline cultures, (mRNA) *Ttr* levels peaked at a BMP4 concentration of 16 ng/ml (Figure 1E). In CPCs, (mRNA) *Msx1* levels increased monotonically (Figure 1F), as reported previously [12], [15]. These findings indicate that the *Ttr* and *Msx1* responses to BMP4 are cell-intrinsic.

### FGF receptor inhibition mimics the effects of exogenous BMP4

The rostral expansion of *Ttr* in BMP4-treated explants suggests that a suppressor of *Ttr* expression exists in the rostral midline. FGFs produced in the RM, particularly FGF8, are candidates for mediating this suppression, as FGF8 has been shown to negatively influence the BMP pathway in the dorsal telencephalon [25], [26]. To test this idea, we treated explants with 100 nM PD173074, a pan-FGF receptor (FGFR) inhibitor [30] (IC_50_ = 21.5 nM, K_d_ = 45.2 nM). These explants displayed rostral *Ttr* expansion reminiscent of that seen in BMP4-treated explants (n = 4/6; Figure 1I); no such changes were seen in control DMSO-treated explants (n = 0/4; Figure 1G). Additionally, placing FGF8-soaked beads adjacent to the endogenous CPE resulted in consistent *Ttr* suppression (n = 8/12 compared to 0/12 BSA-soaked controls; Figure S1D). These results suggest that FGF8, and possibly other rostral FGFs, normally suppress CPE fate and *Ttr* expression. In addition, the similarity between BMP4- and PD173074-induced *Ttr* responses suggests that individual midline cells can respond identically to either increased BMP or reduced FGF signaling. Restricted *Ttr* induction towards the RM also supports a biphasic model for rostral FGF functions in DM development – i.e. rostral FGFs first provide competency for DM fates, then inhibit them [11] – as seen for FGFs in the chick midbrain DM [11], [31].

We then examined *Msx1* expression in PD173074-treated explants. Ectopic *Msx1* induction was less extensive with PD173074 than with BMP4, but like BMP4, PD173074 treatment led to ectopic *Msx1* induction in the cortex but not the midline (n = 7/8; Figure 1H,J). In addition, ectopic *Msx1* expression in cortical regions overlapped with PD173074- and BMP4-treated explants (arrows, Figure 1D and J). These findings reveal an FGFR-mediated suppression of *Msx1* in the cortex, possibly mediated by FGFs expressed by cortical cells, such as FGF2 and FGF1 [20]. They also indicate that cortical cells can respond similarly to either increased BMP or reduced FGF signaling.

To test if FGFR signaling regulates BMP target gene responses at the single cell level, we treated dissociated midline cells or CPCs with PD173074. We found that FGFR inhibition upregulated (mRNA) *Ttr* (Figure 1K) in midline cells and *Msx1* (Figure 1L) in CPCs. This indicated that FGFR signaling, presumably activated by FGFs produced by the cultured cells themselves, inhibits the BMP target genes. Next, we tested whether exogenous FGFs inhibit BMP target genes in dissociated midline cells and CPCs. Given the explant results, FGF8 was used in midline cultures, while CPCs were treated with FGF2. Increasing FGF8 led to decreasing *Ttr* expression in midline cells (Figure 1M). Similarly, FGF2 resulted in concentration-dependent *Msx1* decreases in CPCs (Figure 1N). Thus, FGF-mediated inhibition of BMP target genes is intrinsic to both midline and cortical cells.

**Figure 2:**
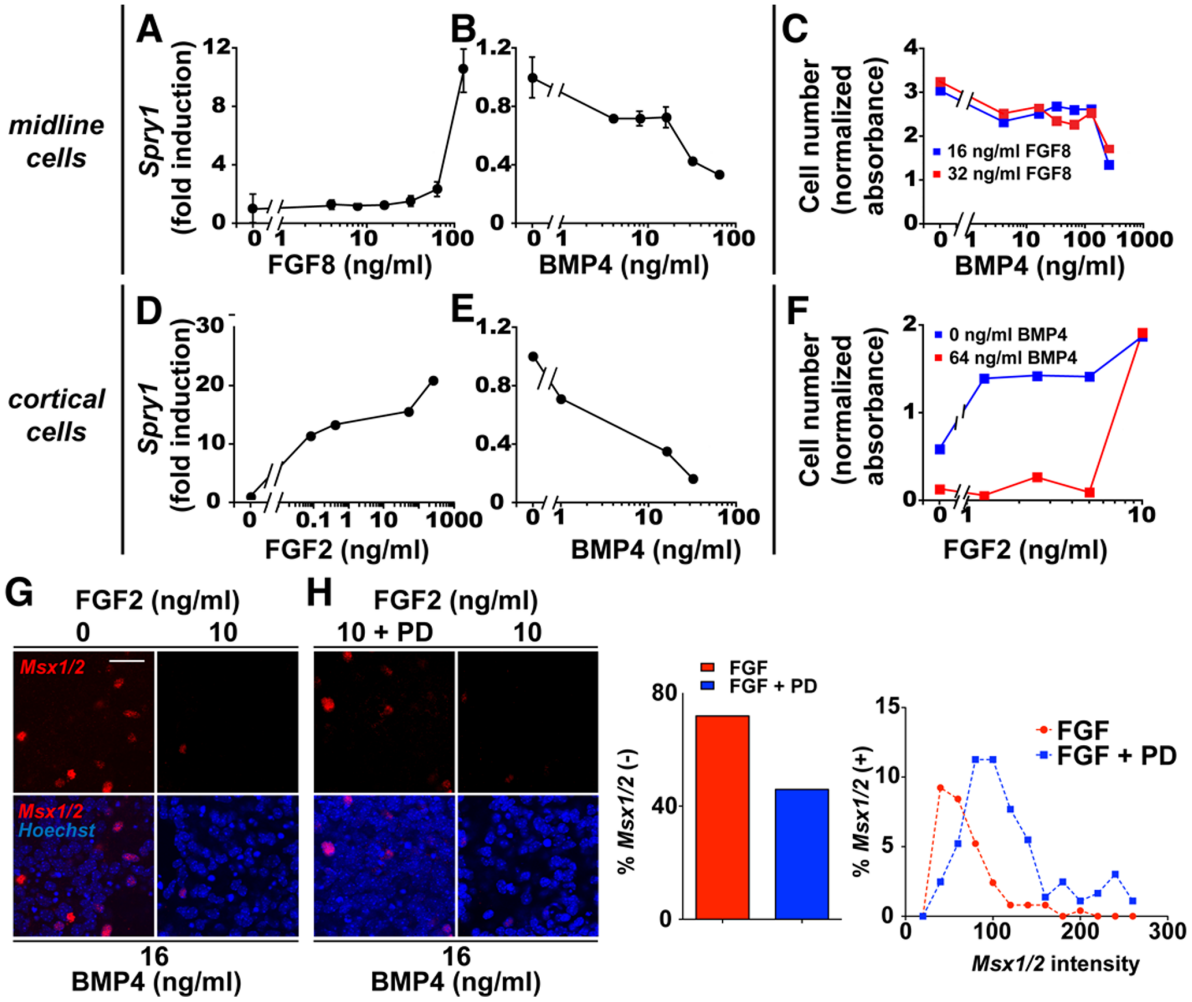
BMP4 inhibits FGF target responses in midline cells and CPCs. (**A,B,D,E**) RT-qPCR on dissociated E12.5 midline cells (A,B) or CPCs (D,E). FGF8 (A) and FGF2 (D) monotonically increase (mRNA) *Spry1* in midline cells and CPCs, respectively. BMP4 downregulates (mRNA) *Spry1* levels in a dose-dependent fashion in midline cells (B) and CPCs (E). (**C**) Cell number (WST1) assay on dissociated E12.5 midline cells treated with FGF8 at 16 ng/ml (blue) or 32 ng/ml (red). These two concentrations, but not 8 ng/ml, led to significant midline cell proliferation (Figure S1E). BMP4 decreases FGF8-driven cell proliferation in a dose-dependent fashion. (**F**) WST1 assay on dissociated E12.5 CPCs. FGF2 increases CPC number with increasing dosage, while BMP4 (64 ng/ml) suppresses the proliferative effect of FGF2. (**G,H**) MSX1/2 immunocytochemistry of E12.5 CPCs. 10 ng/ml FGF2 reduces MSX1/2-positive cell numbers and MSX1/2 levels per cell (G). The FGFR inhibitor PD173074 (100 nM) increases *Msx1/2*-expressing cells and expression levels in CPCs treated with BMP4 (16 ng/ml) and FGF2 (10 ng/ml) (H). PD173074 reduces the percentage of MSX1/2-negative cells (bar graph) and causes a general increase (right shift) in *Msx1/2* expression per cell. Scale bar: 25 um).

To examine FGF-mediated inhibition at the single cell level, we performed immunocytochemistry on CPCs using an anti-MSX1/2 antibody, as done previously to demonstrate ultrasensitivity at the single CPC level in response to BMP4 [12]. *Msx1/2* expression in E12.5 CPCs was examined under three conditions: 1) at low BMP4 concentrations (1.5 ng/ml) with and without FGF2 (10 ng/ml), 2) at mid-level BMP4 concentrations (16 ng/ml) with and without FGF2, and 3) at mid-level BMP4 concentrations and FGF2 with and without PD173074 (100 nM). As expected, *Msx1/2* positivity and expression levels in CPCs were higher at 16 ng/ml than at 1.5 ng/ml BMP4 (Figure S7A). FGF2 addition led to markedly decreased *Msx1/2* expression at both BMP4 concentrations (Figures 2G, S7A), while PD173074 coapplication rescued *Msx1/2* expression (Figures 2H, S7B), with increased expression levels (right-shift) in MSX1/2-positive cells and fewer MSX1/2-negative cells in the presence of PD173074 (Figures 2H, S7B). Thus, FGF-mediated suppression of BMP target responses in CPCs at the population level, as determined by RT-qPCR, also occurs at the level of individual CPCs.

### BMP signaling downregulates FGF target responses

We next investigated whether BMP4 can inhibit FGF responses. We first confirmed that FGF8 and FGF2 positively upregulate the FGF target gene, (mRNA) *Spry1* [22], [32], in dissociated midline and cortical cells, respectively (Figure 2A,D). Correspondingly, the FGFR inhibitors PD173074 and SU5402 decreased endogenous *Spry1* expression in midline cells (Figure S1E). When BMP4 was administered, (mRNA) *Spry1* levels were downregulated in a dose-dependent fashion in both cell types (Figure 2B,E). Thus, BMP4 can downregulate an FGF target gene in dissociated midline and cortical cells. We also treated CPCs with the BMP receptor inhibitor LDN193189 [33] (IC_50_ = 5 nM). The treatment resulted in dose-dependent decreases in (mRNA) *Msx1* levels, while (mRNA) *Spry1* levels increased (Figure S1F). These results with LDN193189 - the converse of those obtained with BMP4 – provide further support that BMP signaling inhibits the FGF target gene *Spry1*.

As an additional test for BMP4 inhibition of the FGF pathway, we examined how BMP4 affected FGF-stimulated cell proliferation [34], [35]. FGF2 is a known mitogen for CPCs in culture [20]. We found that FGF8 also acted as a concentration-dependent mitogen for dissociated midline cells (Figure S1E). When BMP4 was coapplied with FGF2 or FGF8, FGF-induced proliferation decreased in a dose-dependent fashion in both cell types (Figure 2C,F). Thus, BMP4 inhibits FGF-driven proliferation as well as *Spry1* expression. Taken together, the experimental data indicate that BMP and FGF signaling inhibit each other's target responses, and that this mutual or cross inhibition is intrinsic to both midline and cortical cells.

### Models of BMP-FGF cross inhibition

How might such BMP-FGF cross inhibition occur? One possibility is that FGF directly inhibits *Ttr* and *Msx1* and BMP4 directly inhibits *Spry1* (Figure S2A). Inhibition could also occur upstream at the level of signaling pathway components or other genes that themselves regulate target responses (Figure S2A). Such upstream inhibition might lead to feedforward and feedback loops (e.g. Figure S2B) and complicated response dynamics. To investigate the behaviors of such systems, we turned to mathematical modeling (Text S1 for rationale and details). In our single cell models, signaling pathways are represented by extracellular morphogens (FGF and BMP), intermediate signals (FGF and BMP intermediates, or F_I_ and B_I_), and target responses (F_T_ and B_T_). Inhibitory links between the pathways were then introduced, resulting in 81 possible configurations (Figure S3). Models were reduced to ordinary differential equations, with interactions represented by Hill functions [36], then grouped by similarity in their steady-state behaviors and topology (Figure S3, Numerical Methods, Text S1 sections 2 and 3).

**Figure 3:**
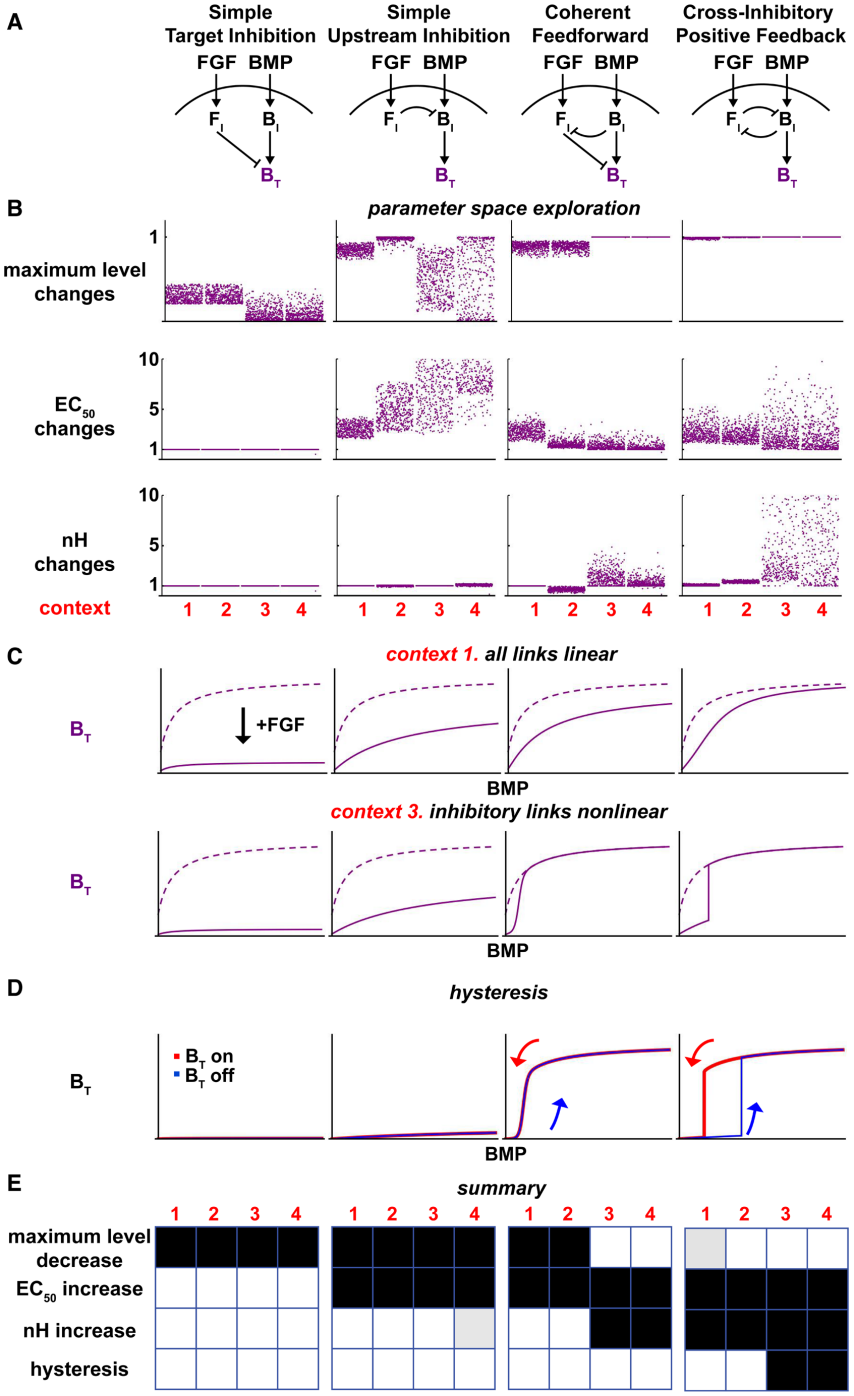
Computational analysis distinguishes BMP-FGF cross inhibition models. (**A**) Representative models of the four classes of BMP-FGF cross inhibition (see text for details). (**B**) Changes in three BMP target (B_T_) response properties (maximum levels, EC_50_, and nH) upon FGF addition under different linearity contexts (in red: 1, all links linear; 2, BMP core pathway nonlinear; 3, inhibitory links nonlinear; 4, all links nonlinear). Each point represents the relative change upon FGF addition for a specific parameter set, and each graph represents 2000 simulations, or 500 per context. (Top row) Maximum levels are maintained with CFF (contexts 3 and 4) and CIPF (all contexts). STI and SUI generally lead to reduced maximum levels. (Second row) Except for STI, EC_50_ values generally increase across models and contexts. (Third row) Only CFF and CIPF increase nH upon FGF addition. Except for a few parameter sets, CIPF consistently increases nH, even under all linear conditions. (**C**) Representative BMP dose-response simulations with and without FGF (solid and dashed lines, respectively) for context 1 (inhibitory strength 88%, nH = 1) and context 3 (inhibitory strength 65%, nH = 4). Upon FGF addition, CFF (context 3 only) and CIPF lead to B_T_ suppression at low BMP concentrations, while B_T_ maximum levels are maintained at high BMP concentrations. This leads to increased EC_50_ values and the emergence of ultrasensitivity. (**D**) BMP dose-response simulations (inhibitory strength 76%, nH = 4) with different initial conditions (B_T_ starts ‘off’ in blue or ‘on’ in red). Based on initial conditions, CIPF produces different B_T_ response curves, thus displaying hysteresis, while STI, SUI, and CFF produce identical curves. (**E**) Summary of modeling results. Grid coloring indicates the presence (black), absence (white), or a less than 10% presence of the property (grey) indicated on the left: e.g. B_T_ maximum levels decrease with the STI model in all contexts and is shown by black boxes for contexts 1, 2, 3, and 4. See Table S4 in Text S1 for summary values, Tables S1, S2, and S3 in Text S1 for parameter values. See also Figure S4.

This grouping resulted in four classes of models described by generalized equations (Table 1): two non-feedback classes, with FGF-to-BMP inhibition occurring at or upstream of B_T_, one feedforward, and one feedback. We refer to these classes as: 1) simple target inhibition (STI), 2) simple upstream inhibition (SUI), 3) coherent feedforward (CFF), and 4) cross-inhibitory positive feedback (CIPF). Representative models that captured the basic response dynamics of each class are shown in Figure 3A (equations 1–4, and Text S1 for modeling rationale). For simplicity, and because our work focuses on BMP targets (B_T_), interaction and nodes that have no influence on B_T_ (e.g. F_T_) are omitted from these depictions. Later, we will argue that the particular selections of inhibitory connections in the representative models are likely to be well justified (see Discussion).

**Table 1:** Generalized equations for each class of models.

Models	Generalized B_T_ solutions
STI	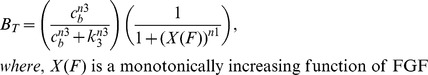
SUI	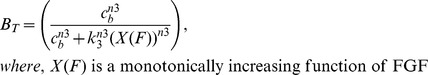
CFF	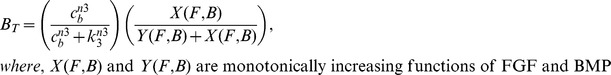
CIPF	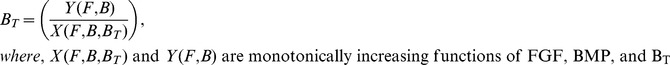

*c_b_* – saturated BMP signal, *k_3_* – half maximal saturation constant for B_T_, *n3* – Hill coefficient for B_I_ activation of B_T_. See also Figure S3 and Text S1 for derivations.

Both CFF and CIPF are known motifs. CFF can provide for a “sign-sensitive delay” that protects outputs against transient activation spikes [37]. CIPF, first identified by Monod as the theory of double bluff [38], is also known as mutual negative feedback [39], double negative feedback [40], or the “toggle switch” motif [36]. It operates during cell fate specification in many developmental systems (e.g [10]). In these systems, CIPF serves to compare two inputs, ultimately turning on targets for the stronger one while turning off those for the weaker input. Depending on the relative strengths of the inputs, CIPF can therefore toggle between two mutually-exclusive sets of target genes.

### The BMP-FGF cross inhibition models generate distinct responses

One way to compare the different models is to examine the dose-response relationships between BMP and its targets (B_T_) in the presence or absence of a fixed amount of FGF. Under these circumstances, three features of the B_T_ response are potentially informative: maximal levels, EC_50_ values, and sensitivity. The sensitivity could be either linear (hyperbolic) or ultrasensitive (sigmoidal) to varying degrees, as quantified by its apparent Hill coefficient, or nH. Changes in these response features were evaluated over a wide range of parameter space and across different “contexts” in which different links within the models were made nonlinear to different degrees (Figures 3B,C, S4, Text S1 sections 3 and 4, Materials and Methods for curve fitting).

While CIPF and STI produced consistent response changes across contexts, CFF and SUI produced more context dependent response changes (Figures 3B,C,E, S4B). Notably, only CFF and CIPF created or enhanced ultrasensitivity (Figure 3B,C,E). CIPF always increased ultrasensitivity even with all links linear and more so with non-linear inhibitory links (Figures 3B,E, S4B). With CFF and CIPF parameters that increased ultrasensitivity, FGF decreased B_T_ levels at low BMP concentrations and had negligible effects on B_T_ at high BMP concentrations. Such selective B_T_ suppression at low BMP concentrations invariably resulted in more sigmoidal dose-response curves with higher EC_50_ values (Figures 3C, S4E). This was the invariant pattern by which ultrasensitivity emerged or increased with CFF and CIPF in the presence of FGF.

### Dependence of CPC ultrasensitivity on FGF signaling supports CFF or CIPF

**Figure 4:**
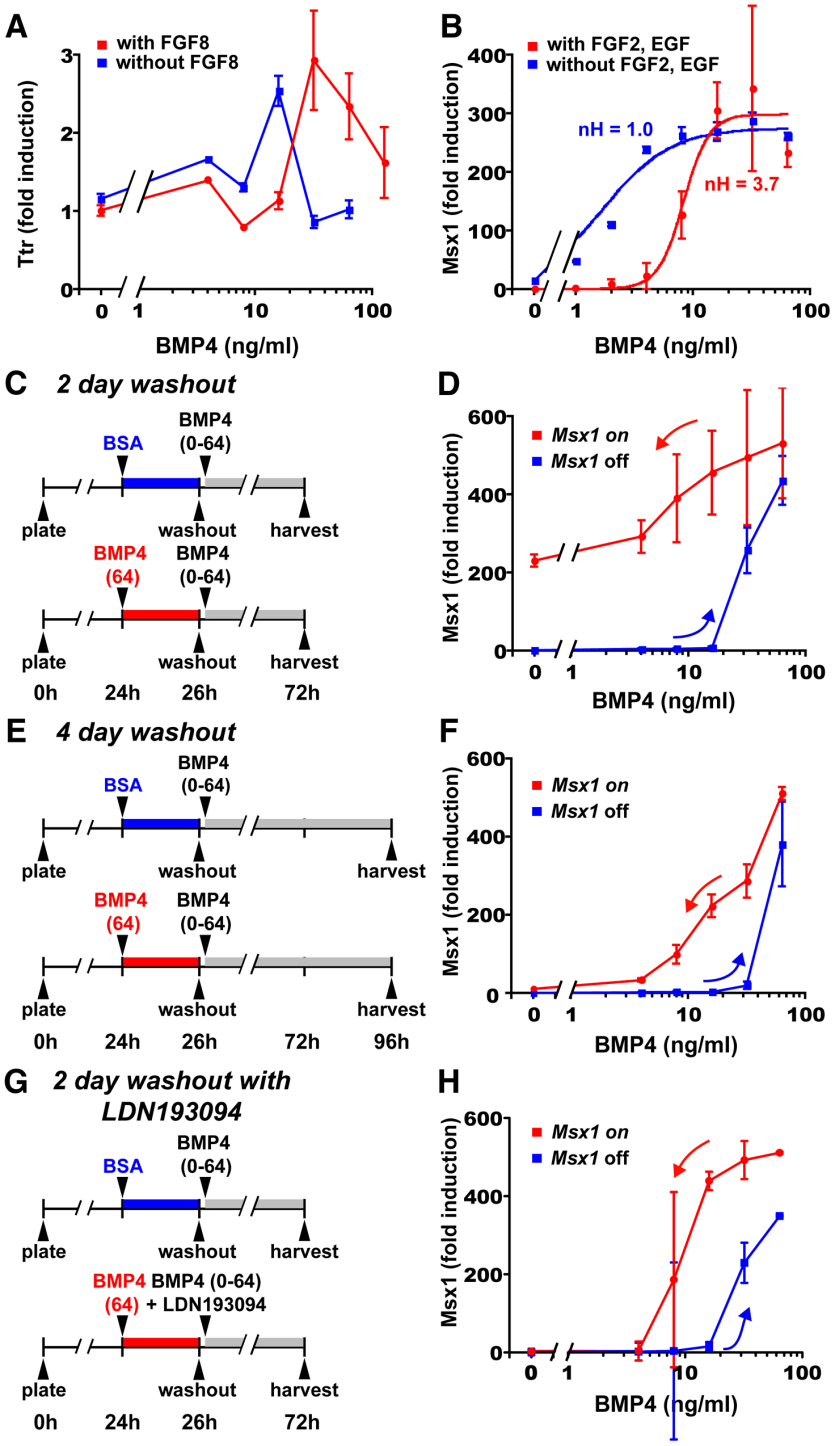
Threshold tuning, ultrasensitivity, and hysteresis experiments implicate CIPF. (**A**) *Ttr* responses of E12.5 midline cells to BMP4 with and without FGF8 (red and blue lines, respectively; RT-qPCR). FGF8 shifts the (mRNA) *Ttr* peak from 16 to 32 ng/ml BMP4, but maximal (mRNA) *Ttr* levels and its apparent ultrasensitivity are unchanged. (**B**) *Msx1* responses of E12.5 CPCs to BMP4 with and without FGF2 and EGF (red and blue lines, respectively, curve fit to a Hill equation; RT-qPCR). *Msx1* responds linearly to BMP4 (nH = 1.0) in the absence of FGF2 and EGF. With FGF2 and EGF, *Msx1* induction becomes ultrasensitive (nH = 3.7) due to its selective suppression at low BMP4 concentrations, as predicted by the CFF and CIPF models. (**C–H**) Washout paradigms and RT-qPCR results for the hysteresis experiments with E12.5 CPCs. For G and H, BMP4 and the BMPR inhibitor LDN193189 were coapplied following BMP4 washout. *Msx1* mRNA levels remain relatively high 2 days after BMP4 washout (D). However, after 4 days (F) or after 2 days in the presence of LDN193189 (H), CPCs initially treated with BSA (blue lines) follow different *Msx1* induction curves from those treated initially with BMP4 (red lines), thus displaying hysteresis. Error bars represent s.e.m.

To distinguish the models, we performed dissociated culture studies. In the absence of FGF8, maximal *Ttr* expression in midline cells occurred at 16 ng/ml BMP4 (Figure 1K). With FGF8 (8 ng/ml), maximal (mRNA) *Ttr* levels did not change, but the EC_50_ increased to ∼31 ng/ml BMP4 (Figure 4A). The effects argue against an STI model, but are consistent with SUI, CFF, or CIPF (Figure 3B,E).

We then evaluated *Msx1* ultrasensitivity in dissociated CPCs. Our previous *Msx1* studies utilized CPCs cultured with FGF2 and EGF [12]. Like FGF2, EGF signaling had an inhibitory effect on *Msx1* in CPCs: EGF downregulated (mRNA) *Msx1* levels, while the EGF receptor inhibitor PD153035 [41] (IC_50_ = 25 pM) produced dose-dependent *Msx1* upregulation (Figure S1G). Notably, EGF is expressed in the antihem and may form a rostro-lateral to caudal-medial gradient [42].

With FGF2 and EGF, *Msx1* induction by BMP4 displayed ultrasensitivity (nH = 3.7; red curve in Figure 4B), as described previously [12]. In the absence of FGF2 and EGF, however, *Msx1* induction followed an ideal hyperbolic curve (nH = 1.0; blue curve in Figure 4B). The dose-response curve with FGF2 and EGF differed in four ways from those without them: 1) *Msx1* mRNA levels were reduced at low BMP4 concentrations, 2) maximal (mRNA) *Msx1* levels at high BMP4 concentrations were unchanged, 3) the EC_50_ increased (1.5 to 8.3 ng/ml), and 4) marked ultrasensitivity emerged. These characteristics precisely matched the CFF (with nonlinear inhibitory links) and CIPF models (compare Figures 3C and 4B).

### Presence of hysteresis implicates CIPF

To further distinguish among models, we tested for a property of positive feedback systems known as hysteresis, a form of cellular memory or bistability [43], [44]. Cells display hysteresis when their dose-response curves differ depending on whether they start in an ‘on’ or ‘off’ state (e.g. whether CPCs start with *Msx1* highly expressed or not, Figure S4D). Importantly, hysteretic responses, unlike irreversible ones [43], turn off after stimulus removal (Figure S4D). We examined all four models for their ability to produce hysteresis or any form of bistability.

The STI, SUI, and CFF models, unlike CIPF, yielded steady state solutions for B_T_ that were amenable to analysis. When tested mathematically, STI, SUI, and CFF always produced identical on- and off-curves for B_T_. However, CIPF - which was examined with simulations and monotone stability analysis [43] - produced different on- and off-curves under certain conditions (Figures 3D, S4E, S5). Thus, amongst the four cross inhibition models, only CIPF is capable of generating hysteresis. It is possible to produce bistable responses with other motifs, such as auto-regulatory feedback, but these are unlikely in this system (see Figure S4E and Text S1 sections 5 and 6 for discussion on feedback and other models).

Further analysis showed that hysteresis occurs only if at least one of the CIPF loop links was nonlinear, and both links were roughly matched in strength (Figure S5B–D, Text S1 section 5). This requirement for a “balanced” CIPF loop can be explained by considering cases in which F_I_ or B_I_ is far stronger than the other. When F_I_ dominates, CIPF reduces to SUI, whereas when B_I_ is too strong, CIPF reduces to the BMP core pathway; and neither SUI nor the BMP core pathway alone can generate hysteresis. A balanced CIPF loop was also required to produce ultrasensitivity, and the magnitudes of increased sensitivity and hysteresis (i.e. size of the hysteresis ‘window’) correlated strongly (Figure S5C–E). Thus, a balanced nonlinear CIPF loop is required for hysteresis and increased ultrasensitivity.

To test for hysteresis, CPCs were cultured for two hours with high BMP4 (64 ng/ml) to induce the *Msx1* ‘on’ state [12], or with BSA to maintain CPCs in the *Msx1* ‘off’ state. After washing out the BMP4 or BSA thoroughly, media alone (no BMP4) or BMP4 at different concentrations (4–64 ng/ml) was reapplied before harvesting CPCs two days later (Figure 4C). In CPCs exposed to BSA, then low BMP4, *Msx1* expression remained low (Figure 4D, blue line). However, in CPCs exposed to high BMP4, then no or low BMP4 after washout, (mRNA) *Msx1* levels remained high (Figure 4D, red line), as previously observed [12]. Since *Msx1* did not turn off after BMP4 removal, *Msx1* appeared irreversible rather than hysteretic. However, another explanation was persistent BMP signaling after washout. Persistent signaling was possible, since slow dissociation of BMP and other TGF-beta molecules from their receptors can lead to prolonged signaling even after free extracellular ligand is removed [45], [46], [47].

To address the possibility of persistent signaling, we modified the above experiment in two ways. First, we cultured the cells for a longer period after washout before harvesting (four days; Figure 4E). In CPCs treated with high BMP4, then no or low BMP4 after washout (0–4 ng/ml), (mRNA) *Msx1* did indeed return to low baseline levels (Figure 4F, red line). Thus, *Msx1* induction was not irreversible. When comparing the CPCs initially treated with BSA (*Msx1*-off) or high BMP4 (*Msx1*-on), (mRNA) *Msx1* levels were higher in the *Msx1*-on CPCs regardless of the BMP4 concentration that was reapplied after washout (compare red and blue lines in Figure 4F). In other words, we observed hysteresis.

For the second modification, we repeated the two-day culture studies, but also included the BMPR inhibitor LDN193189 during and after BMP4 washout to block persistent BMP signaling (Figure 4G). In CPCs initially exposed to high BMP4, LDN193189 caused (mRNA) *Msx1* to return to low baseline levels when no or low BMP4 was reapplied (0–4 ng/ml; Figure 4H, red line). Thus, persistent BMPR signaling contributed to the maintained *Msx1* expression observed initially (Figure 4D, red line). As in the four-day cultures, there were two distinct curves that depended on initial conditions - i.e. whether CPCs were initially *Msx1*-on or *Msx1*-off (compare red and blue lines in Figure 4H). Thus, the response again displayed hysteresis. Collectively, the two lines of evidence for hysteresis strongly implicate CIPF as the mechanism underlying BMP-FGF cross inhibition in CPCs.

### CIPF can generate multiple EC_50_ values for different target genes

**Figure 5:**
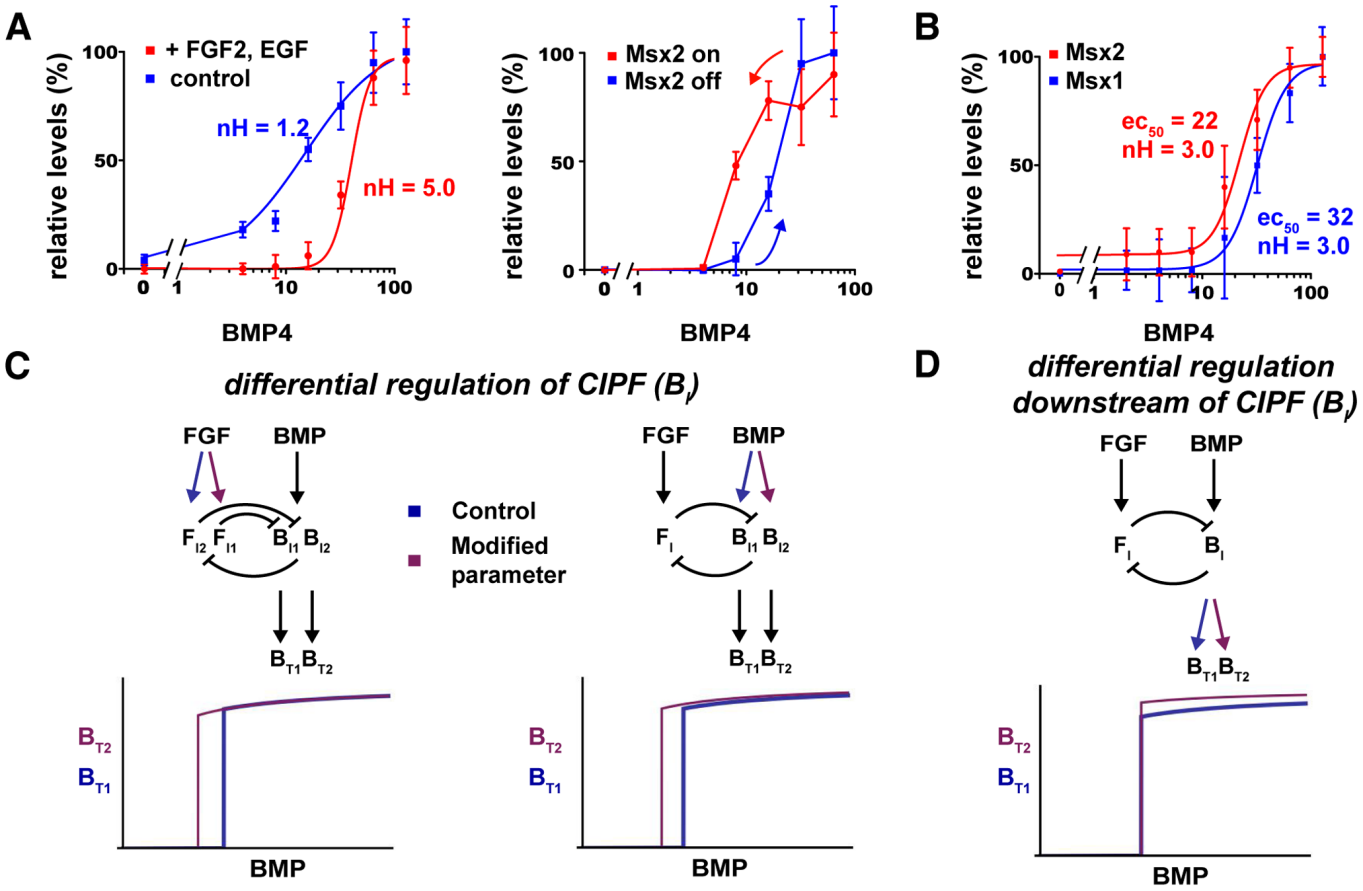
BMP-FGF CIPF leads to distinct *Msx1* and *Msx2* EC_50_ values. (**A,B**) *Msx2* responses in E12.5 CPCs with the same paradigms used for *Msx1* in Figure 3. (A, Left panel) *Msx2* induction by BMP4 alone is linear (blue line, nH = 1.2). With FGF2 and EGF, *Msx2* induction becomes ultrasensitive (red line, nH = 5.0) similar to Figure 3C. (A, Right panel) In the 2-day washout paradigm with BMPR inhibitor LDN193189, CPCs treated initially with BSA (blue) follow a different *Msx2* induction curve compared with those treated initially with BMP4 (red), thus displaying hysteresis. (**B**) *Msx1* (blue) and *Msx2* (red) responses to BMP4 in the same CPCs, with FGF2 and EGF present. *Msx2* has a lower EC_50_ (22 ng/ml BMP4, nH = 3.0) than *Msx1* (32 ng/ml BMP4, nH = 3.0). (**C,D**) CIPF network changes that affect B_T_ EC_50_ values. (C) Networks in which the balance between B_I_ and F_I_ in the CIPF loop is different produce different EC_50_ values. (D) Network changes downstream of the CIPF loop, such as B_I_-to-B_T_ gains, do not shift EC_50_ values. See also Figure S6.

*Msx2*, like *Msx1*, is a pSmad-dependent BMP target gene expressed in the telencephalic DM [16], [48]. In CPCs treated with increasing BMP4, but no FGF2 or EGF, *Msx2* expression was linearly sensitive and increased monotonically (Figure 5A). With FGF2 and EGF, *Msx2* induction by BMP4 was ultrasensitive to a similar degree as *Msx1*. *Msx2* expression was also hysteretic in two-day washout studies in the presence of LDN193189 (Figure 5A).

While *Msx2* and *Msx1* responses were qualitatively similar, the *Msx2* EC_50_ (22 ng/ml BMP4) was significantly lower than that of *Msx1* (32 ng/ml BMP4) in the same cells (Figure 5B). In traditional views of morphogen action, EC_50_ values and border positions are inversely related – e.g. a lower EC_50_ shifts borders away from a morphogen source, thus creating a larger expression domain for a positively-regulated target gene. Published images of the dorsal telencephalon suggest that the *Msx2* domain is indeed larger than the *Msx1* domain [48]. To verify this, we stained adjacent sections from *Msx1* (*Msx1-nlacZ*) embryos which express nuclear *lacZ* in the *Msx1* domain [49], with antibodies that detect lacZ or MSX1/2 proteins. The results suggest that the E10.5 *Msx2* expression domain extends ∼100 µm farther than that of *Msx1* (8 sections from 2 embryos; Figure S6A).

For morphogen thresholds ∼1.5 fold apart (22 vs. 32 ng/ml BMP4 for *Msx2* and *Msx1*, respectively; Figure 5B) and separated by 100 µm, the length scale of an exponential morphogen gradient (the distance over which morphogen concentration falls by 1−*e* ^−1^, or ∼63%) would be ∼270 µm. This value agrees well with the dorsoventral pSmad gradient in the E10.5 dorsal telencephalon [15], whose best-fit exponential curve had a length scale of ∼290 µm (see Materials and Methods). Thus, *Msx1* and *Msx2* EC_50_ values *in vitro* correlate well with their expression borders *in vivo*.

How might CIPF produce different borders or EC_50_ values with the same BMP and FGF gradients? We found that it is possible to produce two distinct EC_50_ values and borders, when BMP and FGF drive two different intracellular CIPF loops with common elements. The two CIPF loops can differ in two ways: either at the level of the CIPF loop - through differential regulation of the intermediates (B_I_ and F_I_) by BMP and FGF or differential loop inhibition between the intermediates - or downstream of the CIPF loop. Our simulations show that the first category produced B_T_ responses with separate EC_50_ values (Figures 5C, S6B–C, Text S1 section 7), while differences downstream of the CIPF loop did not (Figures 5D, S6B,D).

## Discussion

A variety of mechanisms have been proposed to explain how shallow morphogen gradients are converted into sharp borders of gene expression and cell fate. In the dorsal telencephalon, previous work implicated cell-intrinsic BMP-driven ultrasensitivity in this conversion [12]. Here we show that this form of ultrasensitivity arises from cross inhibition between BMP and FGF (and EGF) signaling pathways. Such inhibition manifests as cross-inhibitory positive feedback (CIPF), a mechanism characterized by ultrasensitivity (Figures 3–5, S4, S5), hysteresis (Figures 3, 4, 5, S4, S5) and an ability to generate different thresholds (EC_50_ values) within a gradient (Figures 5, S6).

The complete elimination of *Msx1* and *Msx2* ultrasensitivity in the absence of FGF2 and EGF (Figures 4, 5) indicates that CIPF alone can account for *Msx1* and *Msx2* ultrasensitivity. In midline cells, however, FGF elimination caused a left-shift of the bimodal *Ttr* induction by BMP4 without eliminating ultrasensitivity (Figure 4A). Consistent with this, FGFR inhibition shifted the rostral *Ttr* boundary in forebrain explains, without making a more diffuse boundary or extending expression into the cortex (Figure 1). This implies that mechanisms in addition to CIPF might contribute to sharp *Ttr* borders. The relatively late induction of *Ttr in vivo* (two days after *Msx* genes) and its apparent irreversibility [18] suggest that regulation of *Ttr* is more complex than *Msx* genes, which are direct and immediate BMP targets. The differences between *Ttr* and *Msx1* in the BMP4- and FGFR inhibitor-treated explants also raise the possibility that *Ttr* and *Msx1* are regulated by different FGFs (e.g. FGF8 and FGF2). Although changes in cell composition after two days in culture may contribute to observed findings, previous two-day cultures on more naïve E10.5 CPCs yielded similar ultrasensitive *Msx1* responses [12], and BMP2 (which has very similar effects to BMP4 on CPCs) did not significantly alter cell type ratios in comparably-staged rat CPC cultures in the presence of FGF2 even after eight days [50], [51]. While no single parameter set from the simulations and parameter searches can be considered representative, these modeling techniques enable investigation of complex models such as CIPF and CFF that are not otherwise amenable to analytical techniques, which can be sampled over large areas of parameter space (Tables S1, S2, S3, S4, S5 in Text S1) and cross-validated with other methods (Table 1).

### Molecular basis for BMP-FGF CIPF in the forebrain

BMP-FGF cross inhibtion, particularly specification of mutually exclusive BMP- and FGF-dependent cell fates, has been reported in diverse developmental contexts (Table S6 in Text S1, e.g. [52]). The most well-studied molecular mechanism proposed to explain FGF inhibition of BMP signaling has been the ability of FGF-activated ERK to phosphorylate and trigger the degradation of BMP-activated pSmads [27], [28]. This mechanism, however, is unlikely to account for CIPF in the forebrain for two reasons. First, there is no apparent pSmad ultrasensitivity to BMP in CPCs. *In vivo*, pSmad1/5/8 immunoreactivity declines gradually and smoothly with distance from the DM [15] while MSX1 and MSX2 borders are sharp (Figure S6, [16], [48]). In CPCs *in vitro*, in the presence of FGF2 and EGF, nuclear pSmad levels exhibit a graded relationship to BMP4 dose while *Msx1* and *Msx2* inductions are ultrasensitive (Figures 4, 5, [12]). Ultrasensitivity must then be generated downstream of pSmad. Second, the occurrence of *Msx1* and *Msx2* EC_50_ values at different BMP4 doses suggests a mechanism downstream of pSmad as well (Figure 5B). As *Msx1* and *Msx2* share the pSmad activation pathway, the points of FGF inhibition into the BMP pathway required for separate EC_50_ values (Figure 5C,D) probably lie downstream of pSmad (e.g. a Smad-induced gene or Smad coactivator complex). Although less is known about mechanisms underlying BMP inhibition of FGF signaling, the smooth gradient of phospho-ERK in the developing cortex [24] suggests that this inhibition may similarly occur downstream of ERK activation.

### CIPF and pattern formation

Cross-inhibition (or cross-repression) is, of course, not new in developmental biology (Table S7 in Text S1). Specifically, cross inhibition in the form of a toggle switch (i.e. CIPF) is thought to underlie the generation of sharply-bounded domains of mutually-exclusive cell fates in diverse contexts e.g. [53], [54], [55], including the two well-studied systems of the *Drosophila* embryo and mammalian spinal cord [8], [9]. In these systems, patterning emerges from the collaboration between transcription factor morphogens (Bicoid-Caudal in the syncytial *Drosophila* embryo) or a single extracellular morphogen and a transcriptional network (Sonic Hedgehog in the mammalian spinal cord) [6], [8], [9]. Both architectures contain a toggle switch sub-motif similar to BMP-FGF CIPF, which can generate ultrasensitivity, hysteresis (buffering noise), and multiple sharp borders [6], [8], [9], [56]. The Bicoid-Caudal system also reduces variation in border position and scales borders to tissue size [6], [9], [56], which we reason below should apply similarly to BMP-FGF CIPF.

What is unique about BMP-FGF CIPF – compared to Shh, Bicoid-Caudal, and other defined patterning systems – is that a cellular-level toggle switch is explicitly driven by and dependent on multiple extracellular morphogens. BMP-FGF CIPF therefore provides a direct and explicit link between tissue-level patterning by antagonistic morphogens and cellular-level ultrasensitivity. Unlike the antagonistic Bicoid-Caudal system, BMP-FGF CIPF occurs in a non-syncytial system that may apply broadly to vertebrates, given the prevalence of BMP-FGF cross-inhibition in vertebrate development (Table S6 in Text S1). Furthermore, the current study defines new requirements for CIPF-mediated toggle switches (nonlinearity and loop balance) that likely apply to the *Drosophila* embryo, mammalian spinal cord, and other cross-inhibition systems belonging to the CIPF class of models (Table S7 in Text S1).

**Figure 6:**
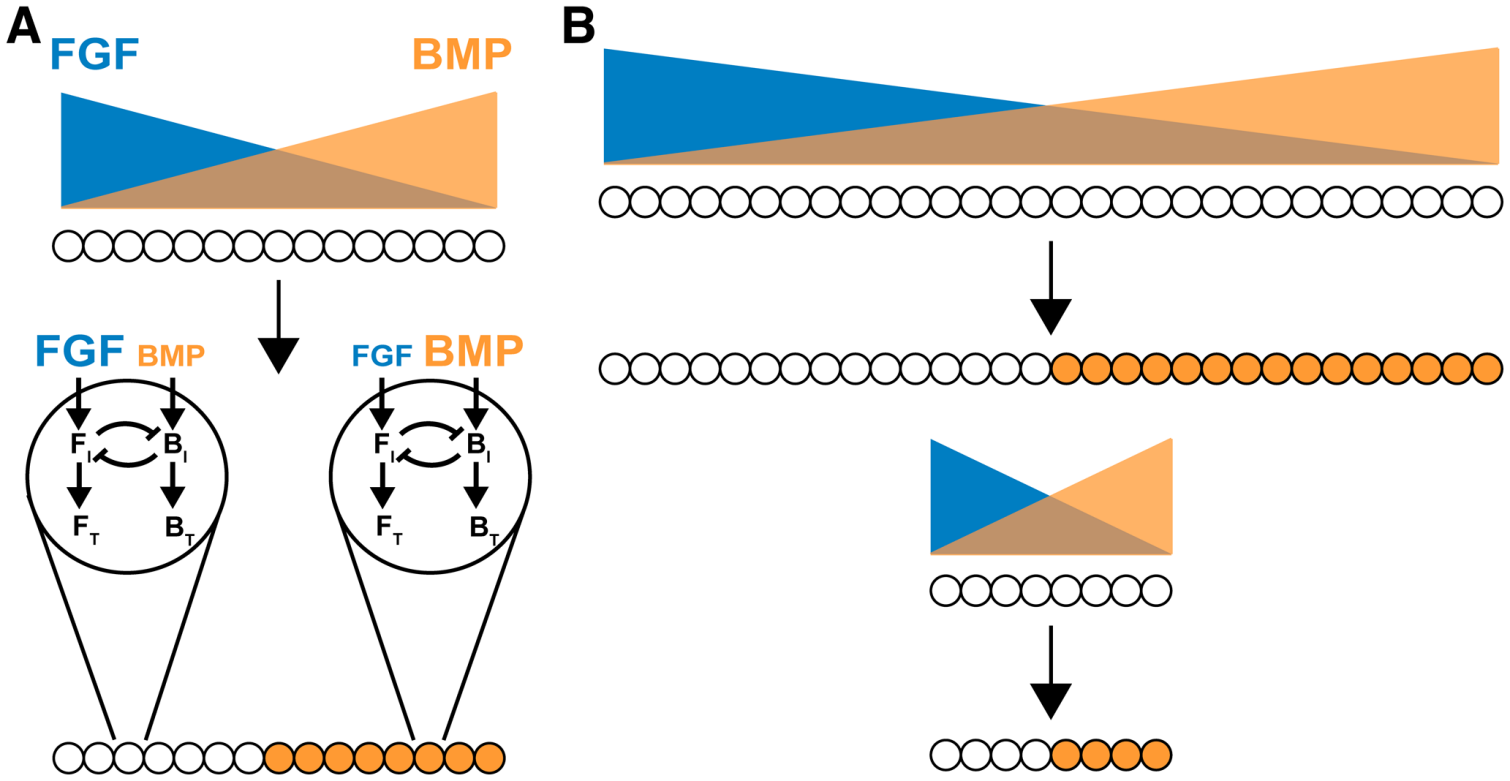
BMP-FGF CIPF in tissue patterning. Schematic of tissues with opposing gradients of BMP (orange) and FGF (blue). Cells on the left experience higher FGF signaling. Cells on the right experience higher BMP signaling. The CIPF toggle switch turns on BMP target genes where BMP>FGF signaling and turns them off where BMP<FGF signaling; forming a border at the BMP-FGF signaling “equivalence point”. The right panels illustrate how CIPF can scale patterns to changes in tissue size. Relative border position is maintained regardless of tissue size as CIPF-dependent borders are determined by BMP-FGF signaling ratio. See Figure S1A for dorsal forebrain schematic.

In the dorsal telencephalon, DM rostral border position would be determined by BMPs interacting with rostral FGF8 and related FGFs (Figures 6A, S1A). Mediolaterally, the same BMPs would interact with FGF2 and FGF1 in the cortex, and possibly with EGF from the antihem [42]. Along both axes, interactions between opposing BMP and FGF/EGF gradients would determine cellular ultrasensitivity thresholds and gene expression borders (Figure 6). These border positions coincide with “equivalence” points – i.e. points at which BMP and FGF signaling (or B_I_ and F_I_) balance each other. The effects of BMP4 or FGFR inhibitors on forebrain explants (Figures 1, S1) can then be understood in terms of shifts in equivalence points.

The ability of morphogen-driven CIPF to specify different equivalence points for different target genes (Figures 5, S6B,C) provides a straightforward mechanism for establishing multiple sharp borders, a general problem in morphogen-mediated patterning [5]. So far, only a few solutions have been discovered for making multiple borders, including the generation of sequential thresholds in protein modification [57], a temporal overshoot mechanism [58], and time-dependent changes in cell competence [4].

A second useful property of CIPF driven by opposing morphogen gradients would be its ability to scale pattern to tissue size (Figure 6B). Pattern scaling is important in development, as size variations naturally arise as functions of time, genetic background, and environment [4]. As others have pointed out, mechanisms that assign positional values based on the ratio between signals emanating from opposing sources, rather than as a function of a single signal, have an inherent tendency to scale [59], [60], although not necessarily with equivalent accuracy at every location. While no studies have investigated scaling of pattern in the forebrain DM, the forebrain provides a promising avenue for future studies. The forebrain grows rapidly during early development, and scaling needs to occur on a spatiotemporal scale. Additionally, mouse and human microcephaly and megalencephaly cases in which patterning appears to be maintained represent a potentially rich area for research into the toggle switch mechanism and brain scaling.

Notably, for morphogen-driven CIPF to specify border positions, it is not necessary for both morphogens to be graded. For example, a BMP gradient superimposed on a uniform FGF field would also produce sharp boundaries at locations corresponding to BMP-FGF equivalence points. This scenario may apply to mediolateral borders in the dorsal telencephalon, where FGF2 production appears to be relatively uniform [20]. Referring to FGF as a “morphogen” in this scenario departs from conventional usage (which presumes a graded distribution), but perhaps in patterning systems driven by collaborations between diffusible signals, such a departure is justified.

## Materials and Methods

### Ethics statement

All animal studies were performed in accordance with protocol # 2001–2304 approved by the Institutional Animal Care and Use Committee (IACUC) of the University of California, Irvine. All experiments were conducted in accordance with protocol # 2001–1024 approved by the Institutional Biosafety Committee (IBC) of the University of California, Irvine. All surgeries were performed on euthanized animals with all efforts made to minimize suffering. Animals were euthanized with carbon dioxide from compressed gas cannisters, with secondary physical method of cervical dislocation to ensure euthanasia.

### Mice

Noon of vaginal plug date was day 0.5; developmental stages were confirmed by crown-rump measurement. CD1 mice (Charles River Laboratories, Wilmington, MA) were used for wild-type studies. *Msx1-nLacZ* mice [49] were mated with CD1 for expression analysis and were genotyped by Xgal staining of limb buds [12].

### Real-time semiquantitative RT-PCR

RNA preps, cDNA syntheses, PCR quality controls, experimental runs, and statistical methods were performed as described previously [13]. Primers and amplicons were verified by melting curve analysis, agarose gel electrophoresis, and tested for amplification efficiency; amplicons were verified by sequencing. cDNA samples were validated for reverse transcription (RT) reaction efficiency and minimal genomic DNA contamination (cDNA/genomic target ratio >10^5^) and run in duplicate or triplicate for 40 cycles; cyclophilin A (CYPA) and 18S reference primers were included in runs (used for normalization to control for variations between wells and cell populations), except for explant studies (CYPA only). Mean, SEM, SD, and *p* values (two sample, two-tailed t-tests) were calculated from cycle threshold (dCt) values (Ct_gene of interest_ – Ct_reference_) and plotted as normalized ddCt values (upregulation is positive and downregulation is negative). In Figures 1, 2, 4, and 5, relative values were normalized to control.

### Dissociated cell cultures

Midline cells and CPCs were isolated and dissociated from E12.5 CD1 dorsal telencephalon as described previously [12], then plated at 50,000 cells/ml (unless otherwise indicated) in defined media [61]. In previous studies [12], we observed ultrasensitivity in E10.5 cells as well as E12.5 cells. As ultrasensitivity is higher in E12.5 cells and border refinement remains ongoing and continues beyond E12.5, this time point is more amenable and practical experimentally. Midline cultures, in which contamination with cortical cells was likely, were analyzed exclusively for midline-restricted genes (*Ttr*, *Msx1*, *Spry1*) to maintain specificity for midline cells. After adhering overnight, human recombinant BMP4 (R&D Systems, Minneapolis, MN), FGF8, FGF2, EGF (R&D Systems or Peprotech, New Jersey, NJ), heparin (Sigma-Aldrich, St. Louis, MO), PD153035, SU5402 (Tocris Bioscience, Ellisville, MO), PD173074 (Pfizer, New York, NY), and/or LDN193189 (Stemgent, San Diego, CA) were added at indicated concentrations. All RNA purifications (Bio-Rad, Hercules, CA) were done 48 hrs after initial BMP4 treatment except for the following experiments: 1) midline cells treated with FGF8 (Figures 1, 2) were harvested 40 hrs after initial treatment, and 2) midline cells treated with SU5402 or PD173074 (Figure S1) were harvested 12 hrs after initial treatment. For washout experiments (Figures 4, 5), 64 ng/ml BMP4 was applied for 2 hrs, then aspirated and washed three times with fresh media containing 20 ng/ml EGF, 10 ng/ml FGF2, and 2 ug/ml heparin, with or without BMP4 or LDN193189; time points correspond to hours after initial BMP4 application. Graphs represent the following numbers of independent cultures: Figures 1E (n = 3), 1F (n = 6), 1L (n = 3), 2A (n = 2), 2B (n = 2), 2D (n = 2), 2E (n = 3), 4A (n = 3), 4B (n = 3), 4D (n = 2), 4F (n = 2), 4H (n = 3), 5A(i) (n = 3), 5A(ii) (n = 3), 5B (n = 4). Note: Figures 4B, 5A, and 5B show independent separate experiments.

### Explant cultures

Dissections were performed as described [12], [13] in ice-cold L-15 with 2% glucose. Dorsal forebrains from embryos were placed ventricular surface down on the dull surface of 8 µm pore polycarbonate membranes (Whatman, Clifton, NJ) floating on DMEM/F-12 with 20% calf serum, sodium pyruvate, nonessential amino acids, and penicillin/streptomycin. After 1 hr, 50 ng/ml BMP4 was added for three days, or 100 nM PD173074 was added for two days (Figure 1). Explants were processed for *Ttr* ISH or X-gal staining. For FGF8 bead studies (Figure S1), heparin acrylic beads (Sigma) were soaked in 10 µl of 100 ng/ml FGF8 or BSA, rinsed briefly in PBS, and placed on explants using pulled flame-polished microcapillary pipettes.

### *In situ* hybridization, histology, and imaging

These were performed and imaged as described previously [13]. Comparative images and intensity measurements, tissue processing, assays, image acquisition, and processing were performed in parallel on sections from comparable rostrocaudal levels with identical camera settings and enhancements. Parallel image enhancements were limited to levels, brightness, and contrast in Photoshop. Unless indicated, presented images are representative of multiple sections from at least two embryos.

### Proliferation assay

Proliferation studies were performed with a WST1 proliferation assay kit (Clontech, Mountain View, CA). Cells were plated in 96 well plates at matched densities from 25,000–50,000 cells/well. 24 hrs post-plating, BMP4, FGF2, FGF8, and/or EGF at the indicated concentrations was added to media (Figure S1). WST1 was added 36 hrs later at a 1∶10 ratio, and spectrophotometer readings taken 3 hrs post-WST1 addition.

### Immunohistochemistry and immunocytochemistry

Immunohistochemistry was performed as described [12]. Primary CPCs were isolated at E12.5, plated in chambers containing FGF2, FGF2+PD173074, and no FGF media. BMP4 was added 24 hours after plating, and then cultured for 48 hours. Cells were fixed in 4% paraformaldehyde/PBS for 15 mins, permeabilized with 0.3% Triton for 5 mins, and washed in PBS. Cells were blocked with 5% BSA at room temperature for 1 hour, followed by primary antibody in 1% BSA and incubated overnight at 4C. PBS washes were followed by secondary antibody in 1% BSA for 2 hours at room temperature, Hoechst counterstaining, mounting with Vectashield. The following antibodies were used: anti-MSX1/2 (mouse monoclonal antibody against chick Msx1/2; 1∶350 dilution; 4G1; Developmental Studies Hybridoma Bank, University of Iowa), and secondary (Alexa555 goat anti mouse); 1∶500 dilution.

### Curve fitting

For Figures 3, 5 and other modeling results, Hill coefficients (nH) and EC_50_ values were obtained by fitting the B_T_ response to a Hill equation using the Mathematica FindFit function. The maximal values were measured by computing the B_T_ response at an extremely high BMP dose to account for asymptotic behavior. For Figure 3B, the plotted points were obtained by dividing the property value (e.g. nH) of the BMP response in the presence of FGF with that in the absence of FGF. 2000 parameter points were simulated, and for each point, all three properties (maximal levels, EC_50_, and nH) were assessed for all four models (STI, SUI, CFF, and CIPF).

In Figures 4 and 5, nH and EC_50_ values were obtained using the curve fitting function of Deltagraph 6.0. The length scale of the pSmad gradient in the dorsal telencephalon was based on previous data [15]. MS Excel software was used to fit the data to an exponential curve with unknown backgound, 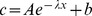, where c is the concentration in ng/ml, *x* the position in µm, *λ* (lambda) the length scale, *A* the highest concentration without *b*, the background.

### Numerical methods

Each of the models was defined by a set of ordinary differential equations. The form of these equations and their rationale is similar to previous modeling approaches [4], [10], [36], [62]. The equations for each model are described briefly, but for modeling details and analysis, please see Text S1.

The four models shown in Figure 3 are represented by the following equations:

1. Simple Target Inhibition (STI) – F_I_ inhibits B_T_:
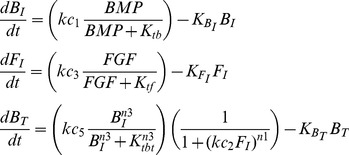
(1)
2. Simple Upstream Inhibition (SUI) – F_I_ inhibits B_I_:
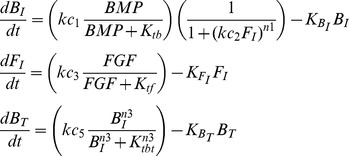
(2)
3. Coherent Feedforward (CFF) – F_I_ inhibits B_T_, B_I_ inhibits F_I_:
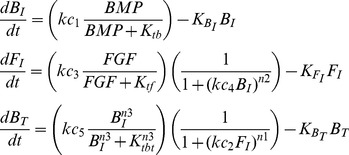
(3)
4. Cross Inhibitory Positive Feedback (CIPF) – F_I_ inhibits B_I_, B_I_ inhibits F_I_:
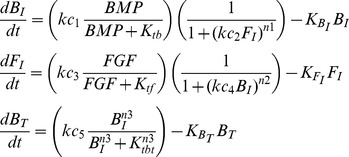
(4)

Explanation of parameters:

*kc_1_, kc_3_, kc_5_* – maximum rates of production (Moles/sec)
*K_tb_, K_tf_, K_tbt_* – half saturation constants of activation (M)
*kc_2_, kc_4_* – half saturation constants of inhibition (1/M)
*K_BI_, K_FI_, K_BT_* – degradation rates (1/s)
*n1, n2, n3* – Hill coefficients
  To reduce the number of parameters and simplify analysis, these equations were non-dimensionalized (see Text S1 equations 7–10), which were then used for modeling and simulations.

## Supporting Information

Figure S1 **Additional evidence for BMPs and GFs affecting DM gene expression.** (**A**) Forebrain schematic (dorsal view, anterior towards the top) showing the DM region where BMP4 is produced (orange), which forms between the two cortical hemispheres (green). FGF2 is produced in the cortex, whereas FGF8 is produced in the rostral midline (blue). (**B**) RT-qPCR of the rostral and dorsal halves of explants. DM-specific genes are upregulated in their endogenous (dorsal) and ectopic domains (rostral) in BMP4-treated explants compared to BSA-treated controls (n = 4 each condition; see also Figure 1c) (**C**) *S*ections of whole-mount explants shown in Figure 1C; ventricular surface down. Compared to BSA-treated explants (control), BMP4-treated explants (50 ng/ml BMP4 added daily for two days) induce *Ttr* ectopically in a 1–2 cell-thick layer that bends toward the ventricle, thus resembling endogenous CPE. (**D**) *In situ* hybridization (ISH), *en face* images of whole mount explants. Compared to control (BSA-soaked) beads (red dashed lines, n = 0/8 explants), FGF8-soaked beads suppress endogenous *Ttr* expression (n = 8/12 explants). (**E**) RT-qPCR and WST1 assays, E12.5 midline cells. The FGFR inhibitors PD173704 (left) and SU5402 (right) downregulate the FGF-target gene *Spry1*. 16 and 32 ng/ml FGF8 (no BMP4 present) increases midline cell numbers, while 8 ng/ml FGF8 had no significant effect. (**F**) RT-qPCR, E12.5 CPCs. SU5402 increases (mRNA) *Msx1* levels, whereas the BMPR inhibitor LDN-193189 reduces (mRNA) *Msx1* levels and increases (mRNA) *Spry1* levels. (**G**) RT-qPCR, E12.5 CPCs. Like FGF, EGF downregulates *Msx1*, and the EGFR inhibitor PD153035 increases (mRNA) *Msx1* levels. Error bars represent standard errors.
(TIF)

Figure S2 **Possible modes of BMP-FGF cross inhibition and behaviors of different CIPF motifs.** (**A**) Three possible ways in which FGFs can inhibit BMP target responses: 1) inhibition directly at the measured target (red), 2) upstream of the target at the level of intermediate messengers (blue), or 3) at the level of second messengers (green). (**B**) One potential BMP-FGF interaction, which generates a cross-inhibitory positive feedback (CIPF) loop. (**C**) Effect of FGF addition on BMP target responses for some of the CIPF motifs shown in Figure S3. See Figure 3 and associated text for description of simulations and contexts. For all CIPF models, adding FGF led to an increase in sensitivity and EC_50_ values, due to suppressed target levels at low BMP concentrations and maintained levels with high BMP. (**D**) An example of stoichiometric inhibition of B_T_. The figure shows the effect of increasing BMP concentration on the concentrations of B_T_ bound to F_T_ and unbound B_T_.
(TIF)

Figure S3 **Classification of BMP-FGF cross inhibition models.** (**A**) After simplifying the BMP and FGF signaling pathways to BMP→B_I_→B_T_ and FGF→F_I_→F_T_, there are 81 potential models of cross inhibition between the two pathways: 9 STI, 18 SUI, 18 CFF, and 36 CIPF. The boxed networks denote the models that were chosen as representative models for the Figure 3. See Figure 3 and associated text for additional details. (**B**) Topologies of possible CFF (top row) and CIPF sub-motifs (bottom row).
(TIF)

Figure S4 **Distinguishing amongst BMP-FGF cross inhibition models.** (**A**) The same four representative models shown in Figure 3. (**B**) Representative BMP dose-response simulations with a nonlinear BMP core pathway (nH = 10 for B→B_I_ or B_I_→B_T_) and with or without FGF (solid or dashed lines, respectively). See Figure 3 and Table S2 in Text S1 for inhibitory link values and other details. (**C**) B_T_ responses with all links linear (context 1), but with increasing inhibitory strengths (95, 97, and 98% represented by increasing line thickness). Other parameters match those in Figure 3C. Increasing inhibitory strength leads to increased ultrasensitivity with CIPF (nH = 2, 2.5, and 3), but not with CFF or the other models, which remain linearly sensitive. (**D**) Schematics of different types of memory; red and blue lines indicate responses that start either on or off, respectively. From left to right – 1) no memory, in which responses do not depend on starting condition; 2) hysteresis, in which responses depend on starting condition, but can return to 0; and 3) irreversibility, in which the response, once ‘on’, never returns to 0. (**E**) Comparison between CIPF (top row) and auto-regulatory positive feedback (bottom row), with increasing feedback strength from left to right. Increasing CIPF feedback strength increases its bistability window, but never produces irreversibility, as B_T_ can always returns to 0. Auto-regulatory positive feedback can generate irreversibility, depending on feedback strength.
(TIF)

Figure S5 **CIPF inhibitory links need to be balanced to produce ultrasensitivity and hysteresis.** (**A**) The CIPF loop with its parameters for strength (*p*) and linearity (*n*). (**B**) Hysteresis, monotone systems analysis. Hysteresis occurs (green dots) when CIPF inhibitory link strengths (left) or nonlinearities (right) are roughly matched. (**C**) Hysteresis, simulation analysis (varied across a wider fold range to those used for Figure 3). Hysteresis simulations similarly require that link strengths or nonlinearities are roughly matched. (**D**) Ultrasensitivity, simulation analysis (same parameters as those used to examine hysteresis). Like hysteresis, ultrasensitivity increases mostly occur when CIPF inhibition strengths or nonlinearities are roughly balanced. (**E**) Comparison of ultrasensitivity and hysteresis increases (bistability window size) for the parameter sets used in Figure 3B. Ultrasensitivity and hysteresis increases are highly correlated (r = 0.93 for context 3, r = 0.92 for context 4).
(TIF)

Figure S6 **Ability of CIPF to generate different EC_50_ values and expression domains.** (**A**) Immunohistochemistry of adjacent coronal sections of E10.5 *Msx1-nlacZ* dorsal telencephalon. The *Msx*1 expression domain detected with anti-lacZ antibody (left) is smaller than the *Msx1+Msx2* expression domain detected with an anti-MSX1/2 antibody (right) by ∼200 µm (∼100 µm per side). Scale bar, 0.2 mm. (**B**) Parameter explorations of B_T_ maximum level and EC_50_ values with two CIPF networks that are different only in terms of links or degradation rates that influence B_T_, B_I_, or F_I_; a value of 1 indicates that the EC_50_ or maximum level is same for both networks. Changes to parameters that affect B_T_ do not change EC_50_ values, while changes to parameters that affect B_I_ and F_I_ in the CIPF loop can produce multiple EC_50_ values. (**C,D**) Continuation of Figure 5C,D: CIPF network changes that produce shifts in B_T_ EC_50_ values. Simulated B_T_ response curves represent unchanged (blue) ‘vs’ modified (purple) CIPF networks. (**C**) Networks in which the balance between B_I_ and F_I_ is changed shift the EC_50_. These shifts occur upon changing the gain or strength of F_I_-to-B_I_ inhibition, B_I_-to-F_I_ inhibition, or the degradation rate of B_I_. (**D**) Network changes downstream of the CIPF loop, such as changes in B_T_ degradation rates, do not shift EC_50_ values.
(TIF)

Figure S7 **BMP4 activates and FGF2 inhibits** ***Msx1/2*** **expression in CPCs at the single cell level.** MSX1/2 immunocytochemistry of E12.5 CPCs. (**A**) With increased BMP4 concentration, more cells express *Msx1/2*. FGF2 (10 ng/ml) markedly reduces *Msx1/2* expression by BMP4 at 1.5 or 16 ng/ml. Scale bar: 0.1 mm. (**B**) The FGFR inhibitor PD173074 (100 nM) increases *Msx1/2* expression in CPCs treated with BMP4 (16 ng/ml) and FGF2 (10 ng/ml). (**C**) Paradigm for the immunocytochemistry single cell experiment. CPCs were plated and treated with media containing FGF, PD+FGF, or no FGF for 24 hours, at which point BMP4 at 1.5 or 16 ng/ml was added. They were then cultured for 48 hours, fixed, labeled for *Msx1/2*, then Hoechst counterstained.
(TIF)

Text S1 **Supplementary information text.** Provides (mostly) mathematical and computational analyses that support the main text. Contents include:
Tables S1, S2, S3, S5 listing parameter values of simulations in Figures 3, 5, S4, and S5.Table S4: a summary of parameter exploration results shown in Figure 3.Table S6: examples of BMP-FGF inhibitory interactions in development.Table S7: examples of toggle switches in development.Supplementary Numerical Methods and Results
Mathematical representation of biological interactionsMathematical models of BMP-FGF mutual inhibitionGenerating and classifying models of BMP-FGF mutual inhibitionParameter explorations to distinguish BMP-FGF mutual inhibition modelsParameters that enable hysteresis and ultrasensitivityContrasting different mechanisms that generate bistabilityParameter explorations of CIPF subnetworks that can produce multiple EC_50_ values
(DOC)
