# Supplementary figures and images for: A BMP-FGF Morphogen Toggle Switch Drives the Ultrasensitive Expression of Multiple Genes in the Developing Forebrain
Source: PLoS Comput Biol. 2014 Feb 13;10(2):e1003463. doi: 10.1371/journal.pcbi.1003463 (PMC3923663; doi:10.1371/journal.pcbi.1003463)

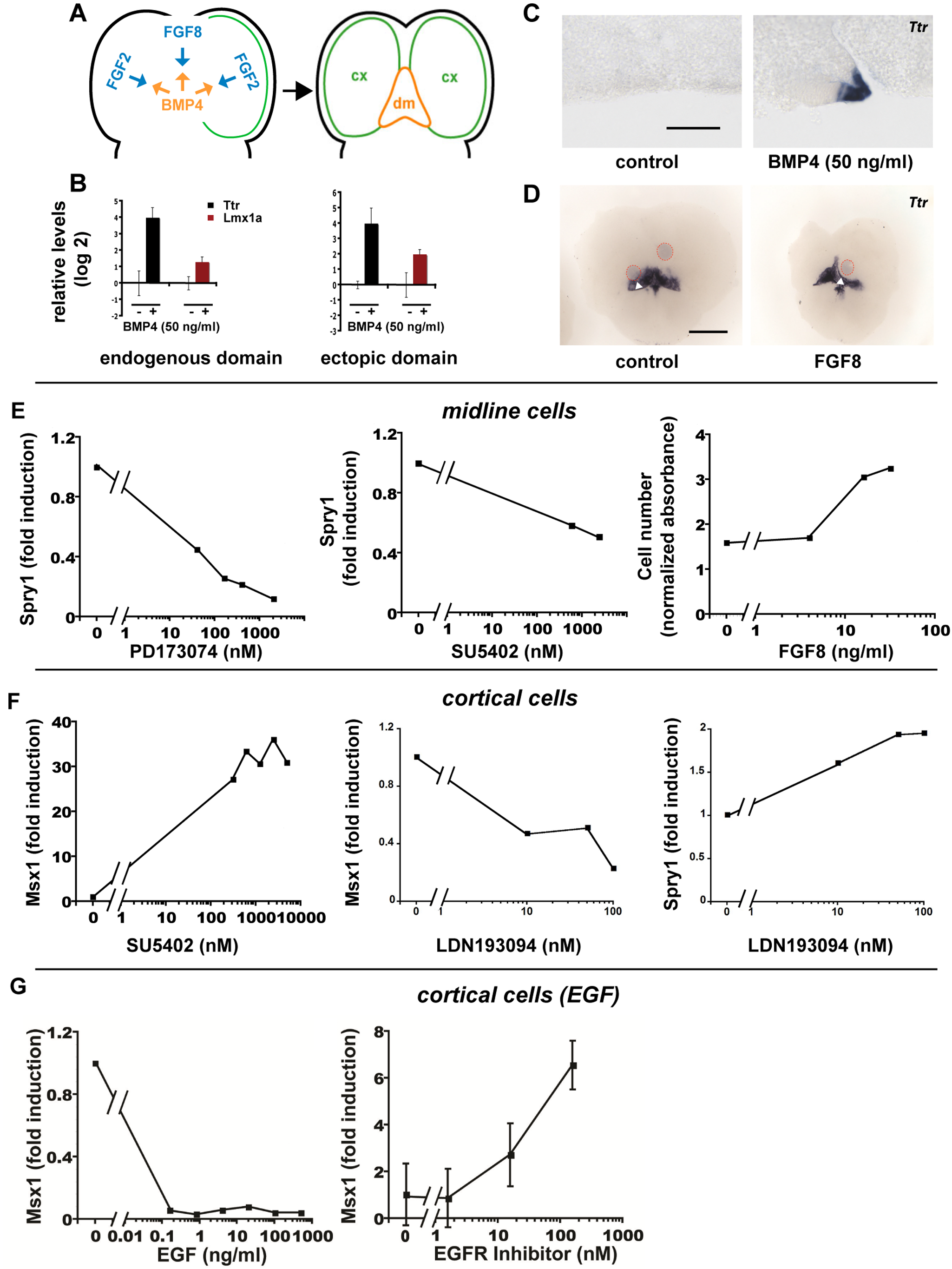

Supplement: Figure S1 — Additional evidence for BMPs and GFs affecting DM gene expression. (A) Forebrain schematic (dorsal view, anterior towards the top) showing the DM region where BMP4 is produced (orange), which forms between the two cortical hemispheres (green). FGF2 is produced in the cortex, whereas FGF8 is produced in the rostral midline (blue). (B) RT-qPCR of the rostral and dorsal halves of explants. DM-specific genes are upregulated in their endogenous (dorsal) and ectopic domains (rostral) in BMP4-treated explants compared to BSA-treated controls (n = 4 each condition; see also Figure 1c) (C) Sections of whole-mount explants shown in Figure 1C; ventricular surface down. Compared to BSA-treated explants (control), BMP4-treated explants (50 ng/ml BMP4 added daily for two days) induce Ttr ectopically in a 1–2 cell-thick layer that bends toward the ventricle, thus resembling endogenous CPE. (D) In situ hybridization (ISH), en face images of whole mount explants. Compared to control (BSA-soaked) beads (red dashed lines, n = 0/8 explants), FGF8-soaked beads suppress endogenous Ttr expression (n = 8/12 explants). (E) RT-qPCR and WST1 assays, E12.5 midline cells. The FGFR inhibitors PD173704 (left) and SU5402 (right) downregulate the FGF-target gene Spry1. 16 and 32 ng/ml FGF8 (no BMP4 present) increases midline cell numbers, while 8 ng/ml FGF8 had no significant effect. (F) RT-qPCR, E12.5 CPCs. SU5402 increases (mRNA) Msx1 levels, whereas the BMPR inhibitor LDN-193189 reduces (mRNA) Msx1 levels and increases (mRNA) Spry1 levels. (G) RT-qPCR, E12.5 CPCs. Like FGF, EGF downregulates Msx1, and the EGFR inhibitor PD153035 increases (mRNA) Msx1 levels. Error bars represent standard errors. (TIF) [file pcbi.1003463.s001.tif]

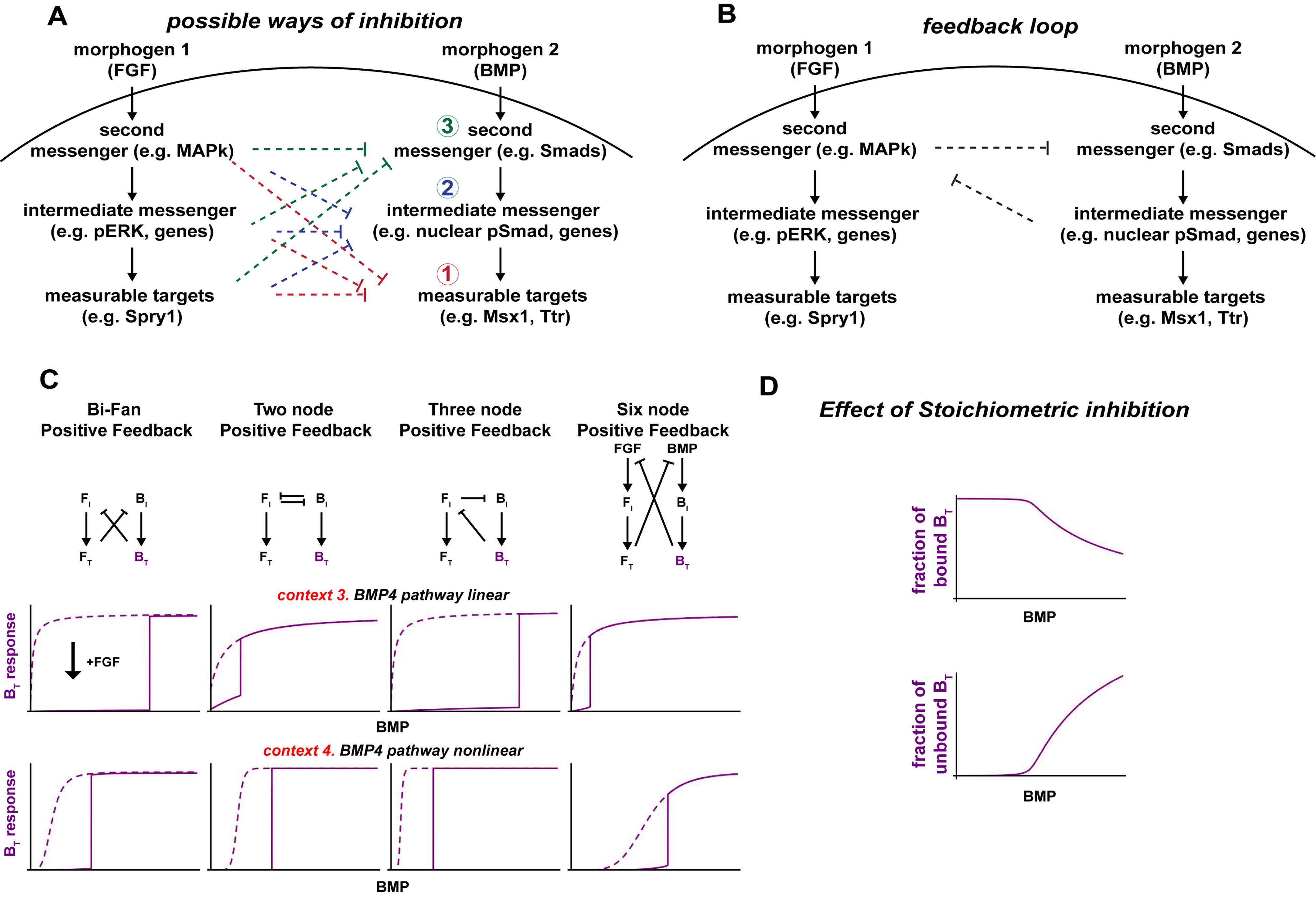

Supplement: Figure S2 — Possible modes of BMP-FGF cross inhibition and behaviors of different CIPF motifs. (A) Three possible ways in which FGFs can inhibit BMP target responses: 1) inhibition directly at the measured target (red), 2) upstream of the target at the level of intermediate messengers (blue), or 3) at the level of second messengers (green). (B) One potential BMP-FGF interaction, which generates a cross-inhibitory positive feedback (CIPF) loop. (C) Effect of FGF addition on BMP target responses for some of the CIPF motifs shown in Figure S3. See Figure 3 and associated text for description of simulations and contexts. For all CIPF models, adding FGF led to an increase in sensitivity and EC50 values, due to suppressed target levels at low BMP concentrations and maintained levels with high BMP. (D) An example of stoichiometric inhibition of BT. The figure shows the effect of increasing BMP concentration on the concentrations of BT bound to FT and unbound BT. (TIF) [file pcbi.1003463.s002.tif]

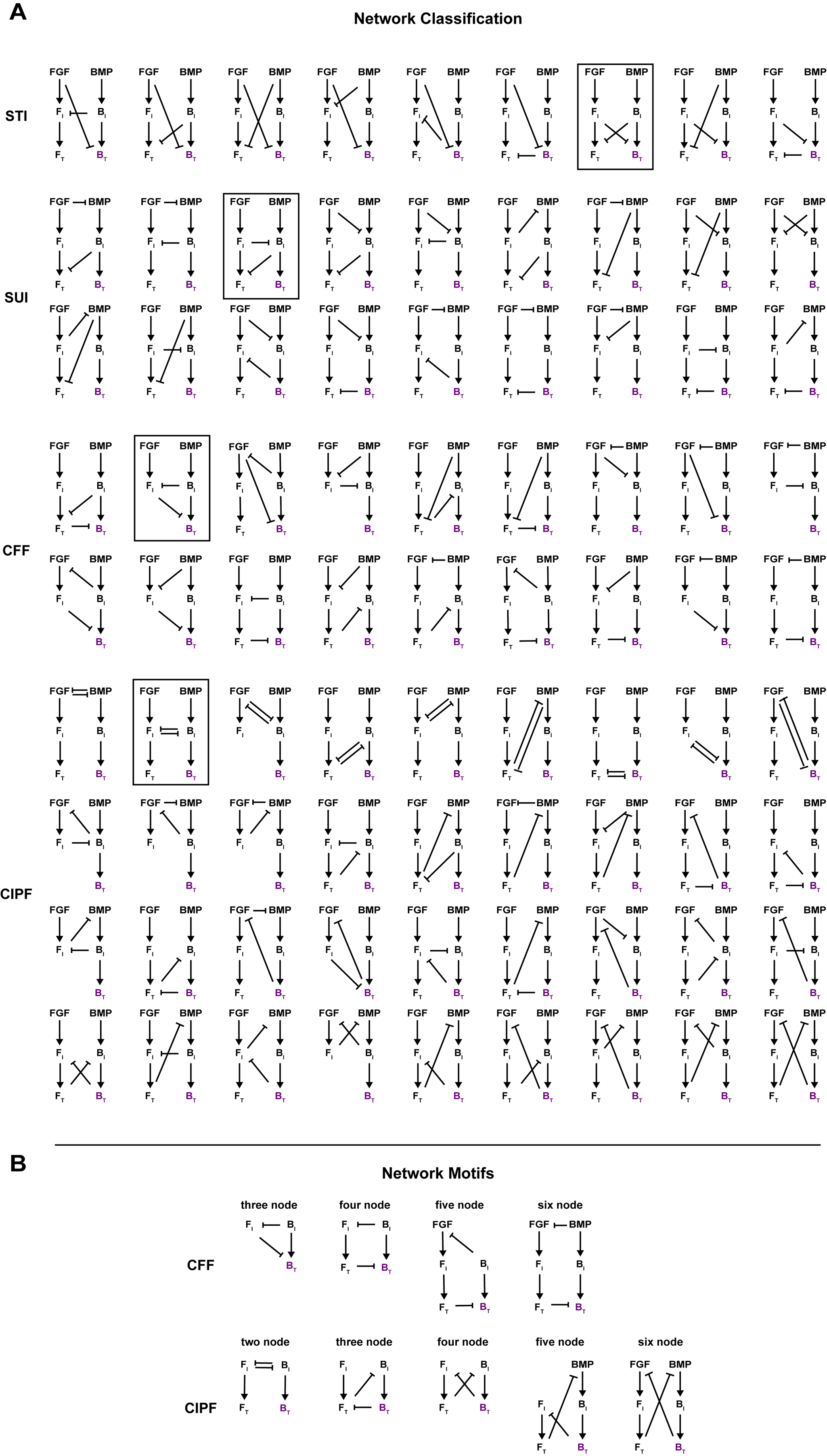

Supplement: Figure S3 — Classification of BMP-FGF cross inhibition models. (A) After simplifying the BMP and FGF signaling pathways to BMP→BI→BT and FGF→FI→FT, there are 81 potential models of cross inhibition between the two pathways: 9 STI, 18 SUI, 18 CFF, and 36 CIPF. The boxed networks denote the models that were chosen as representative models for the Figure 3. See Figure 3 and associated text for additional details. (B) Topologies of possible CFF (top row) and CIPF sub-motifs (bottom row). (TIF) [file pcbi.1003463.s003.tif]

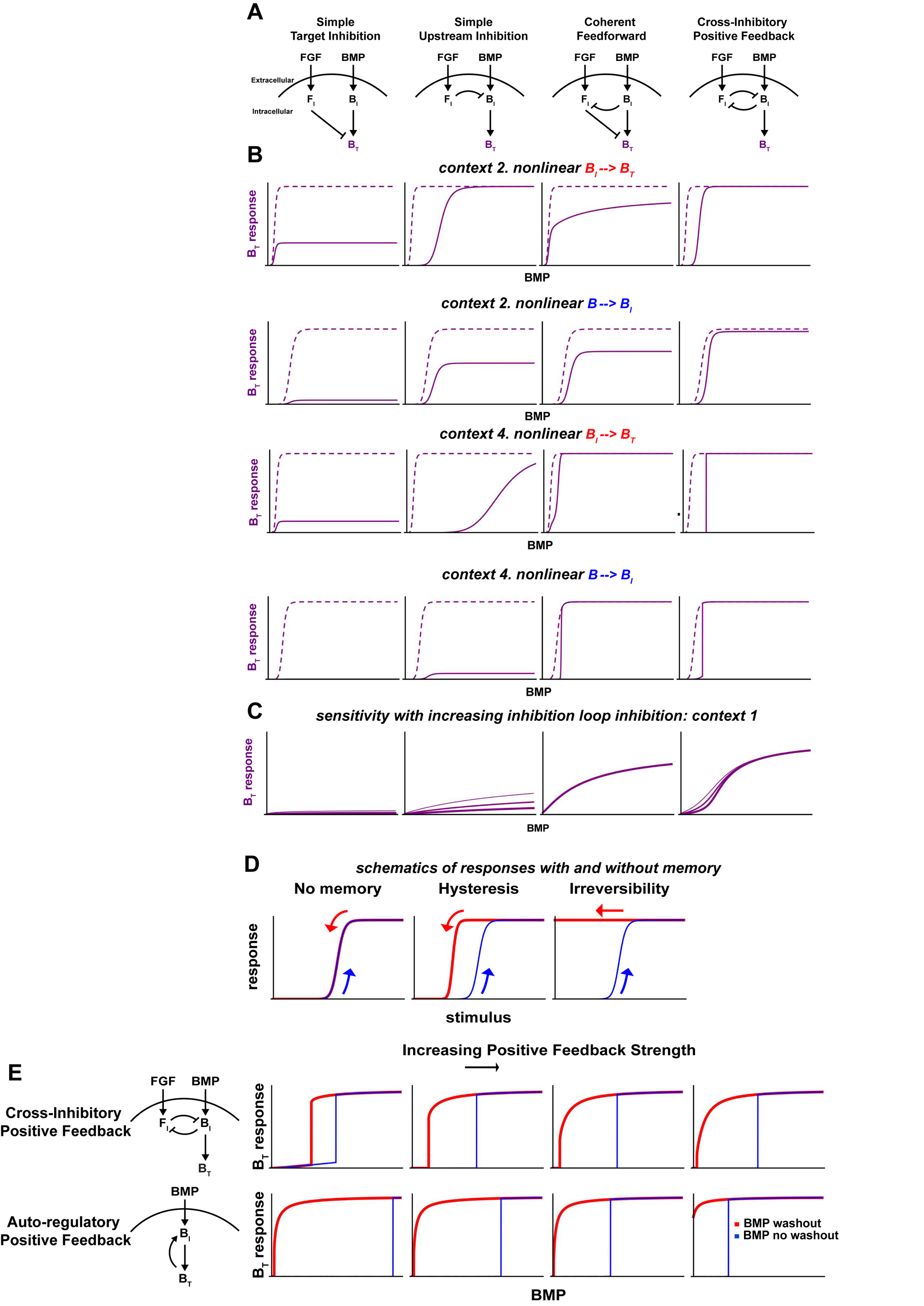

Supplement: Figure S4 — Distinguishing amongst BMP-FGF cross inhibition models. (A) The same four representative models shown in Figure 3. (B) Representative BMP dose-response simulations with a nonlinear BMP core pathway (nH = 10 for B→BI or BI→BT) and with or without FGF (solid or dashed lines, respectively). See Figure 3 and Table S2 in Text S1 for inhibitory link values and other details. (C) BT responses with all links linear (context 1), but with increasing inhibitory strengths (95, 97, and 98% represented by increasing line thickness). Other parameters match those in Figure 3C. Increasing inhibitory strength leads to increased ultrasensitivity with CIPF (nH = 2, 2.5, and 3), but not with CFF or the other models, which remain linearly sensitive. (D) Schematics of different types of memory; red and blue lines indicate responses that start either on or off, respectively. From left to right – 1) no memory, in which responses do not depend on starting condition; 2) hysteresis, in which responses depend on starting condition, but can return to 0; and 3) irreversibility, in which the response, once ‘on’, never returns to 0. (E) Comparison between CIPF (top row) and auto-regulatory positive feedback (bottom row), with increasing feedback strength from left to right. Increasing CIPF feedback strength increases its bistability window, but never produces irreversibility, as BT can always returns to 0. Auto-regulatory positive feedback can generate irreversibility, depending on feedback strength. (TIF) [file pcbi.1003463.s004.tif]

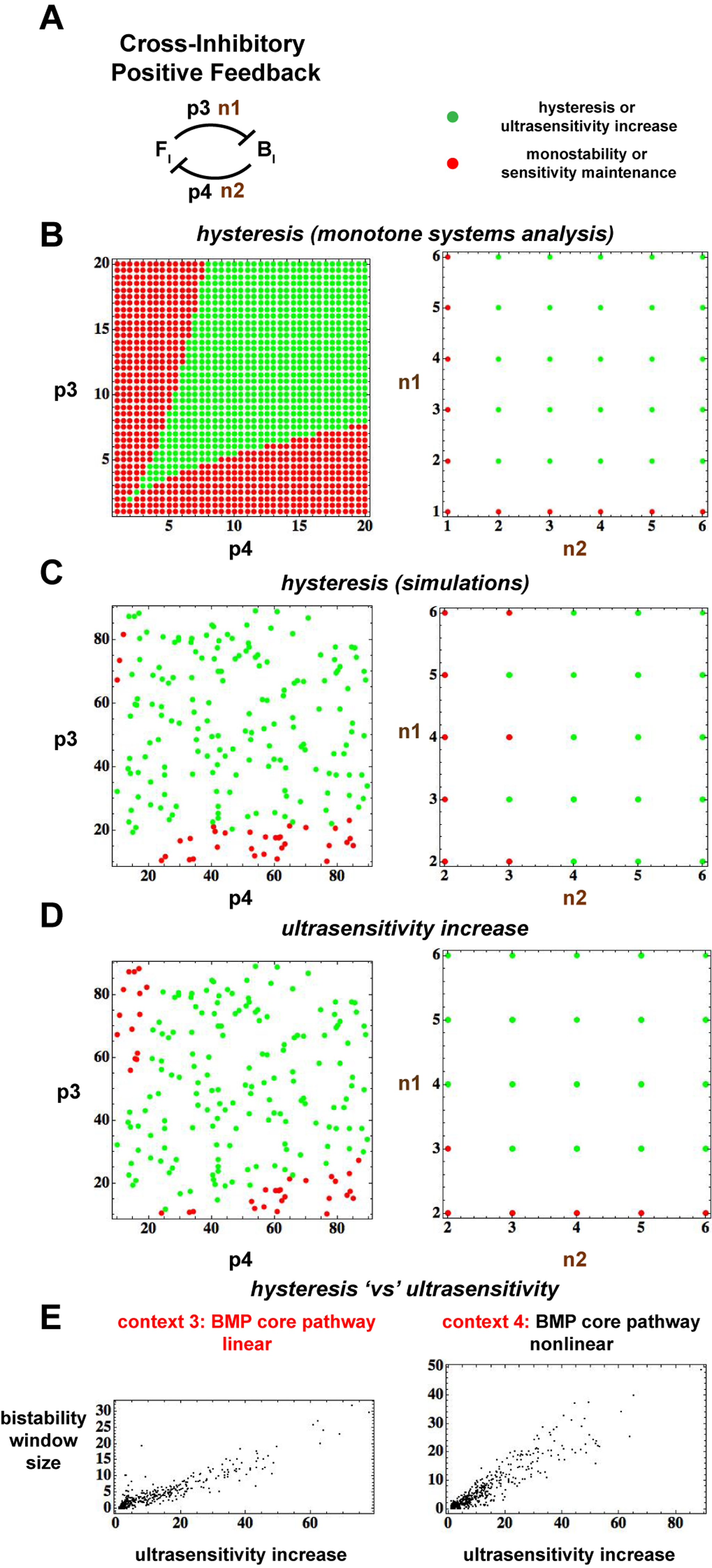

Supplement: Figure S5 — CIPF inhibitory links need to be balanced to produce ultrasensitivity and hysteresis. (A) The CIPF loop with its parameters for strength (p) and linearity (n). (B) Hysteresis, monotone systems analysis. Hysteresis occurs (green dots) when CIPF inhibitory link strengths (left) or nonlinearities (right) are roughly matched. (C) Hysteresis, simulation analysis (varied across a wider fold range to those used for Figure 3). Hysteresis simulations similarly require that link strengths or nonlinearities are roughly matched. (D) Ultrasensitivity, simulation analysis (same parameters as those used to examine hysteresis). Like hysteresis, ultrasensitivity increases mostly occur when CIPF inhibition strengths or nonlinearities are roughly balanced. (E) Comparison of ultrasensitivity and hysteresis increases (bistability window size) for the parameter sets used in Figure 3B. Ultrasensitivity and hysteresis increases are highly correlated (r = 0.93 for context 3, r = 0.92 for context 4). (TIF) [file pcbi.1003463.s005.tif]

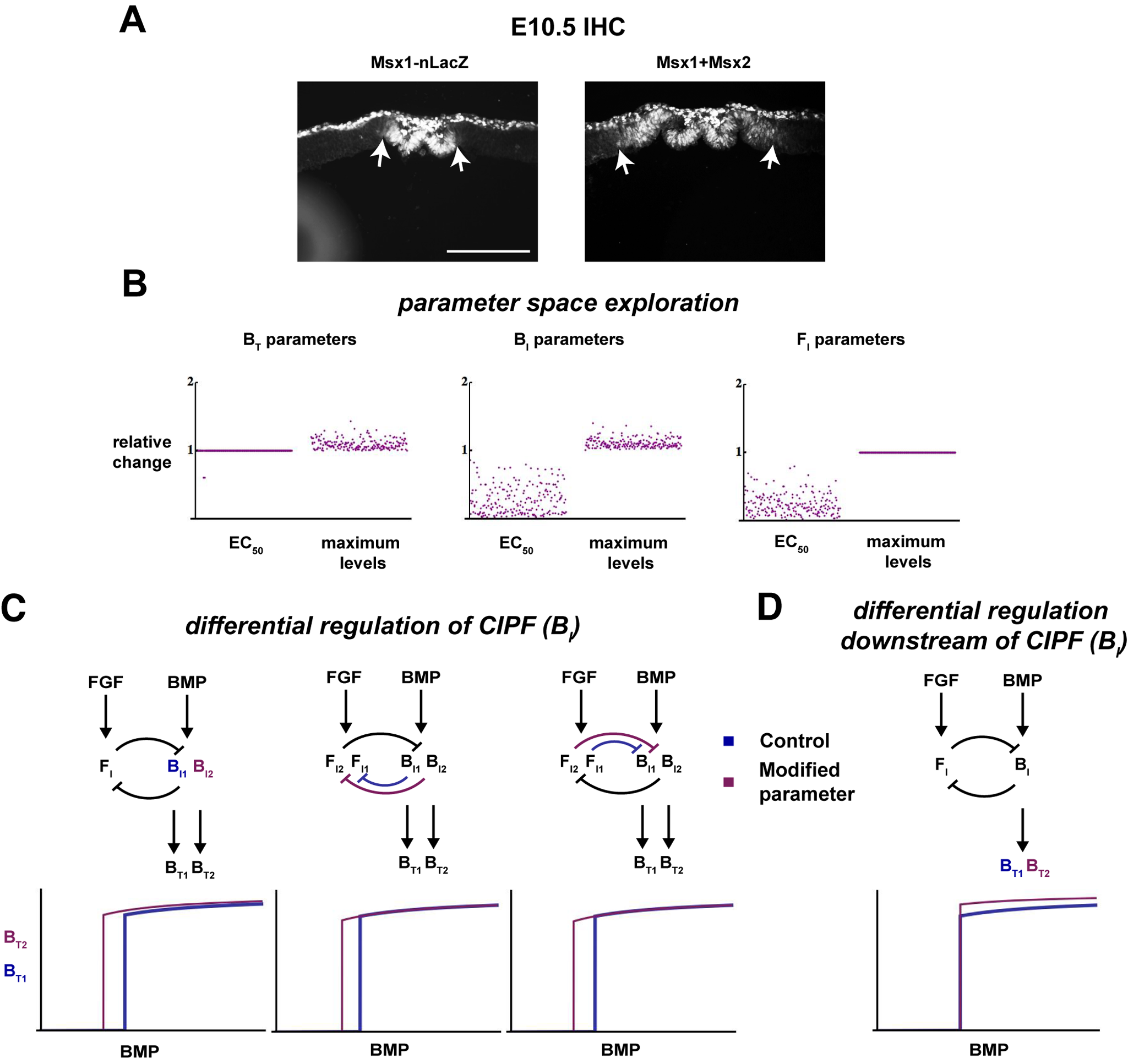

Supplement: Figure S6 — Ability of CIPF to generate different EC50 values and expression domains. (A) Immunohistochemistry of adjacent coronal sections of E10.5 Msx1-nlacZ dorsal telencephalon. The Msx1 expression domain detected with anti-lacZ antibody (left) is smaller than the Msx1+Msx2 expression domain detected with an anti-MSX1/2 antibody (right) by ∼200 µm (∼100 µm per side). Scale bar, 0.2 mm. (B) Parameter explorations of BT maximum level and EC50 values with two CIPF networks that are different only in terms of links or degradation rates that influence BT, BI, or FI; a value of 1 indicates that the EC50 or maximum level is same for both networks. Changes to parameters that affect BT do not change EC50 values, while changes to parameters that affect BI and FI in the CIPF loop can produce multiple EC50 values. (C,D) Continuation of Figure 5C,D: CIPF network changes that produce shifts in BT EC50 values. Simulated BT response curves represent unchanged (blue) ‘vs’ modified (purple) CIPF networks. (C) Networks in which the balance between BI and FI is changed shift the EC50. These shifts occur upon changing the gain or strength of FI-to-BI inhibition, BI-to-FI inhibition, or the degradation rate of BI. (D) Network changes downstream of the CIPF loop, such as changes in BT degradation rates, do not shift EC50 values. (TIF) [file pcbi.1003463.s006.tif]

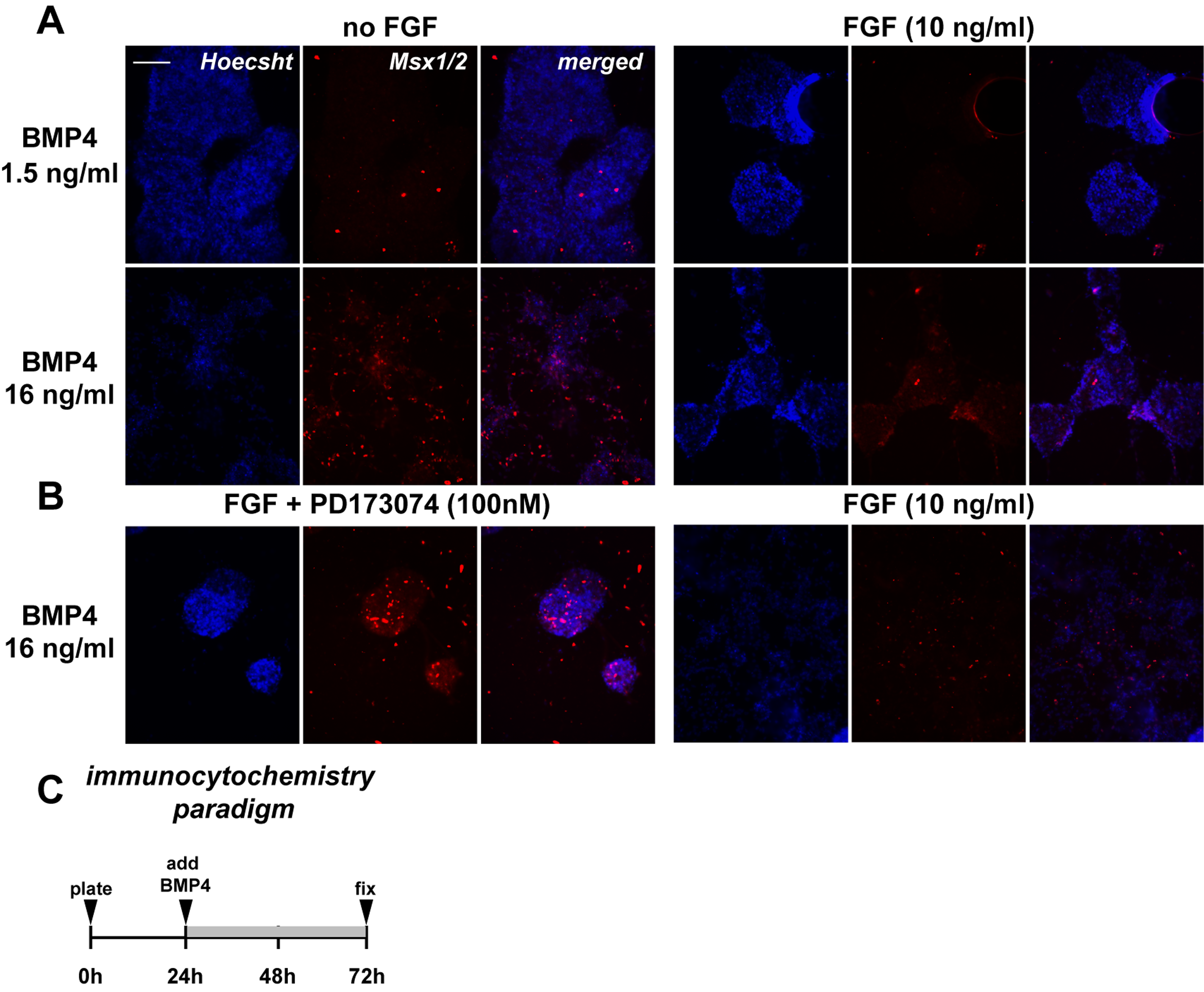

Supplement: Figure S7 — BMP4 activates and FGF2 inhibits Msx1/2 expression in CPCs at the single cell level. MSX1/2 immunocytochemistry of E12.5 CPCs. (A) With increased BMP4 concentration, more cells express Msx1/2. FGF2 (10 ng/ml) markedly reduces Msx1/2 expression by BMP4 at 1.5 or 16 ng/ml. Scale bar: 0.1 mm. (B) The FGFR inhibitor PD173074 (100 nM) increases Msx1/2 expression in CPCs treated with BMP4 (16 ng/ml) and FGF2 (10 ng/ml). (C) Paradigm for the immunocytochemistry single cell experiment. CPCs were plated and treated with media containing FGF, PD+FGF, or no FGF for 24 hours, at which point BMP4 at 1.5 or 16 ng/ml was added. They were then cultured for 48 hours, fixed, labeled for Msx1/2, then Hoechst counterstained. (TIF) [file pcbi.1003463.s007.tif]
